# High-throughput brain activity mapping and machine learning as a foundation for systems neuropharmacology

**DOI:** 10.1038/s41467-018-07289-5

**Published:** 2018-12-03

**Authors:** Xudong Lin, Xin Duan, Claire Jacobs, Jeremy Ullmann, Chung-Yuen Chan, Siya Chen, Shuk-Han Cheng, Wen-Ning Zhao, Annapurna Poduri, Xin Wang, Stephen J. Haggarty, Peng Shi

**Affiliations:** 1 Department of Biomedical Engineering, City University of Hong Kong, 999077 Kowloon, Hong Kong SAR China; 2 Department of Biomedical Science, City University of Hong Kong, 999077 Kowloon, Hong Kong SAR China; 3 Chemical Neurobiology Laboratory, Center for Genomic Medicine, Massachusetts General Hospital, Department of Neurology, Harvard Medical School, Boston, MA 02114 USA; 4 Epilepsy Genetics Program and F.M. Kirby Neurobiology Center, Boston Children’s Hospital, Department of Neurology, Harvard Medical School, Boston, MA 02115 USA; 5 Shenzhen Research Institute, City University of Hong Kong, 518057 Shenzhen, China

## Abstract

Technologies for mapping the spatial and temporal patterns of neural activity have advanced our understanding of brain function in both health and disease. An important application of these technologies is the discovery of next-generation neurotherapeutics for neurological and psychiatric disorders. Here, we describe an in vivo drug screening strategy that combines high-throughput technology to generate large-scale brain activity maps (BAMs) with machine learning for predictive analysis. This platform enables evaluation of compounds’ mechanisms of action and potential therapeutic uses based on information-rich BAMs derived from drug-treated zebrafish larvae. From a screen of clinically used drugs, we found intrinsically coherent drug clusters that are associated with known therapeutic categories. Using BAM-based clusters as a functional classifier, we identify anti-seizure-like drug leads from non-clinical compounds and validate their therapeutic effects in the pentylenetetrazole zebrafish seizure model. Collectively, this study provides a framework to advance the field of systems neuropharmacology.

## Abstract (web summary)

A major goal in neuropharmacology is to develop new tools to effectively test the therapeutic potential of pharmacological agents to treat neurological and psychiatric conditions. Here, authors present an in vivo drug screening system that generates large-scale brain activity maps to be used with machine learning to predict the therapeutic potential of clinically relevant drug leads.

## Introduction

Treatment rather than cure is generally the rule for most central nervous system (CNS) disorders, with many options only providing limited or partial relief^1^. Despite tremendous efforts to elucidate the molecular mechanisms of CNS disorders at the level of specific membrane receptors, ion channels, and signaling pathways, our understanding of the pathophysiology of these disorders remains incomplete^2^. Many clinically effective pharmacological treatment strategies for CNS disorders are the result of serendipitous discoveries, and often affect multiple pathways through diverse functional mechanisms making it difficult to deconvolute the molecular mechanisms underlying efficacy^1,3^. For example, topiramate, an anticonvulsant used broadly for focal and generalized seizures as well as migraine, is known to bind to multiple targets, including voltage-gated sodium channels, high-voltage-activated calcium channels, γ-aminobutyric acid (GABA) receptors, α-amino-3-hydroxy-5-methyl-4-isoxazolepropionic acid (AMPA) receptors and other biogenic amine receptors^3^. In an attempt to address the limitations of the existing CNS pharmacopeia, current CNS drug discovery strategies are often limited by their reliance on overly simplified experimental systems, such as isolated biochemical binding tests and in vitro cell-based assays^4^. While convenient and high-throughput, these systems do not recapitulate the in vivo complexity of the CNS, and are often limited in their ability to predict therapeutic outcomes in the context of human disease biology^5^. Therefore, to advance the development of next-generation pharmacological agents, it is necessary to establish novel paradigms that can monitor and evaluate the complex brain function using multiplexed physiological phenotypes^6,7^. Although drug development efforts using small whole organisms, such as *C. elegans* and zebrafish^8^, make it possible to perform drug screens without prior molecular knowledge of the lead chemicals, the readouts are typically limited to behavioral analysis and morphological phenotyping^6,7,9,10^. Furthermore, these assays suffer from the disadvantage that they fail to correlate a drug’s therapeutic effects directly to physiological changes in the CNS of the model organism. In contrast, whole-brain imaging of small animals, such as zebrafish, provides an attractive means of bridging the gap between large-scale cellular activity and behavioral responses^11,12^. These data can be used to develop drug-screening platforms based purely on complex functional phenotypes^13,14^. To date, an assay of this type has not been demonstrated for CNS drug discovery beyond the testing of a limited number of agents over a small parameter space of dose and time.

In this study, we describe a high-throughput, in vivo drug-screening strategy that combines automated whole-brain activity mapping (BAMing) with computational bioinformatics analysis. Unlike traditional drug screens in relatively simple models^4,15,16^, our strategy utilizes functional brain physiology phenotypes derived from live, nonanesthetized zebrafish that have been treated with compounds of interest as an input for predicting the therapeutic potential of novel bioactive compounds. This technology relies on an autonomous robotic system capable of manipulating awake zebrafish larvae for rapid microscopic imaging of their brains at the level of cellular resolution^13,14^, which allows for rapid assessment of action potential firing across a whole zebrafish brain; as a result, a large number of whole-brain activity maps (BAMs) can be acquired for a compound library.

This study was performed in two parts. The first part employed a training set of 179 clinical drugs to generate information-rich BAMs; the intrinsic coherence among the BAMs for drugs in the training set was determined by a consensus clustering algorithm. Certain BAM clusters were further found to be statistically associated with the drugs’ therapeutic categories as determined by the World Health Organization (WHO) Anatomical Therapeutic Chemical (ATC) classification system. In the second part of the study, a strategy employing machine learning was used to build a functional classifier along with a ranking mechanism to predict the potential therapeutic uses of compounds based upon their similarity to clinically used drugs. Using this machine learning strategy, we highlight the successful prediction of compounds with antiepileptic activity in zebrafish behavioral models from a library of 121 nonclinical compounds, and provid insights that may facilitate development of next-generation antiepileptic agents with novel mechanisms of action.

## Results

### High-throughput brain activity mapping (HT-BAMing) technology

**Fig. 1:**
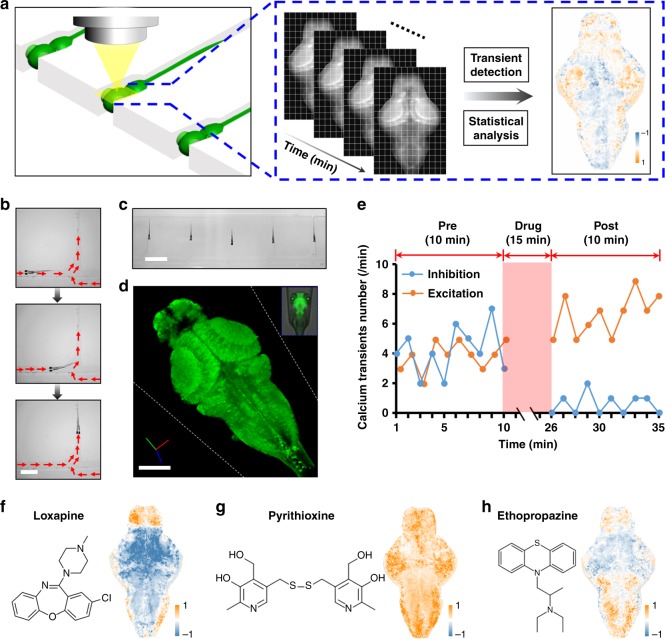
High-throughput brain activity mapping (HT-BAMing) in zebrafish larvae. **a** Illustration of the HT-BAMing technology. **b** Image sequences showing the autonomous larva loading and immobilization in the microfluidic chip. Red arrows indicate the direction of the bulk flow. Scale bar, 2 mm. **c** Immobilization of multiple awake larvae in a microfluidic chip with a dorsal-up orientation. Scale bar, 5 mm. **d** Representative brain image of a larva (from *elavl3*:GCaMP5G transgenic line) trapped in the system. Inset shows the merged image of the larva trapped in the chip. The white dotted line indicates the trapping channel. Scale bar, 100 µm. **e** Representative curves showing the change of calcium fluorescence fluctuation in selected neuronal cells from a zebrafish brain. The sample showing inhibition was treated with the typical antipsychotic loxapine, while the sample data showing excitation follows treatment with pyrithioxine. **f**−**h** T-score BAMs showing the regulation of brain physiology induced by different CNS drugs including **f** loxapine, a dibenzoxazepine-class typical antipsychotic; **g** pyrithioxine, a synthetic, dimeric, disulfide bridged, derivative of vitamin B6 (pyridoxine) with reported psychostimulant/nootropic activity; and **h** ethopropazine, a phenothiazine derivative with anticholinergic, antihistamine, and antiadenergic activities used as an antiparkinsonian medication

Advances in microscopy techniques^17^ and the development of novel calcium-sensitive fluorescent reporters^11^ have enabled recording of brain-wide activity in larval zebrafish with single-cell resolution^12^. Here, changes in the fluorescence of calcium-sensitive fluorophores provides an established proxy for imaging of neuronal activity^11,12,18^. However, the challenges posed by handling of zebrafish larvae prohibit the wide application to large-scale analysis, and as a result the throughput for these experiments is quite limited and can be greatly improved by robotic automation and microfluidic systems^19,20^. With the goal of investigating whole-brain CNS physiology on a large scale, we developed an autonomous system capable of orienting and immobilizing multiple awake, nonanesthetized animals for high-throughput recording of brain-wide neuronal activity. This system was based on our recent development of a microfluidic chip that utilizes hydrodynamic force to trap, position, and orient zebrafish larvae^13,14^ (Fig. 1a−c). The processing throughput was further enhanced by a system for larvae loading and transportation that employs digitally controlled syringe pumps, electromagnetic valves and video detection to enable automatic feeding of larvae into the microfluidic chip (Supplementary Fig. 1). In this study, all larvae were loaded with a dorsal-up orientation to facilitate brain imaging from above. The use of transgenic zebrafish (*elavl3*:GCaMP5G) with a genetically encoded calcium indicator (Fig. 1d) allowed for real-time whole-brain imaging, and subsequent analysis of drug-induced changes in neuronal activity (Supplementary Fig. 1d). To generate the functional BAM of a larva in response to a 15-min period of chemical perfusion, or treatment, each larva was imaged over a 10-min period before and after treatment (Fig. 1e). Readings from multiple focal planes along the *Z*-axis (ventral direction) were acquired, and the accumulated number of calcium transients were derived from the fluorescence fluctuations of each *Z*-plane with a lateral resolution of 15.21 µm^2^. The difference in calcium transient counts between the post- and pretreatment periods was calculated and projected (by summing up along the *Z*-axis) to a two-dimensional surface to construct a BAM that reflects changes in brain activity and physiology of an individual larva in response to treatment (Fig. 1a, e). For each compound, BAMs from five individual larvae were acquired following treatment with a 10 µM dose. To avoid false-positive or false-negative errors potentially introduced by variation among different individuals, the five BAMs for each compound were statistically compared by T-score test at every 15.21 µm^2^ unit across the whole projection surface to extract the brain regions significantly regulated by compound treatment. In addition, a significance score was assigned to each unit to form a T-score brain activity map (T-score BAM) unique to each compound (Supplementary Fig. 2). In the control larvae, (dimethyl sulfoxide) DMSO treatment resulted in white-noise-like T-score BAMs (*n* = 50) (Supplementary Fig. 3).

The HT-BAMing technology presented here enabled large-scale acquisition and rapid analysis of physiologic data and zebrafish brain function. Prior to commencing these studies, it was unclear, however, whether the BAM data from larval zebrafish was sufficiently resolved and information-rich to reflect the complex therapeutic effects and changes in CNS physiology caused by exposure to a mechanistically diverse collection of clinically used drugs. To address this concern, the HT-BAMing technique was applied to a library of 179 clinically used CNS drugs curated to include a variety of distinct mechanisms of action and known therapeutic uses (Supplementary Data 1). Drug treatments did not affect the health of the animals, as ~95% of larvae remained alive and healthy when released from the system. The majority of the drugs tested (92%) did, indeed, induce acute changes in zebrafish brain function with reproducible BAM patterns and, accordingly, T-score BAMs unique to that drug (Fig. 1f−h, Supplementary Fig. 4 and 5). For example, loxapine, a typical antipsychotic drug, resulted in increased neural activity in the forebrain but marked decrease in calcium transients elsewhere in the brain (Fig. 1f, Supplementary Fig. 4 and 5), whereas pyrithioxine, a psychostimulant, increased activity brain-wide (Fig. 1g), and ethoproprazine, used to treat extrapyramidal symptoms in patients with Parkinson’s disease, induced robust upregulation in neural activity only in the forebrain and hindbrain (Fig. 1h). Collectively, these results suggest that BAMs provide a spatially and functionally encoded phenotype directly associated with therapeutic potential of different (or different types of) CNS drugs. To investigate this further, the assay was expanded to test a larger collection of bioactive compounds.

### HT-BAMing-based functional drug screen

**Fig. 2:**
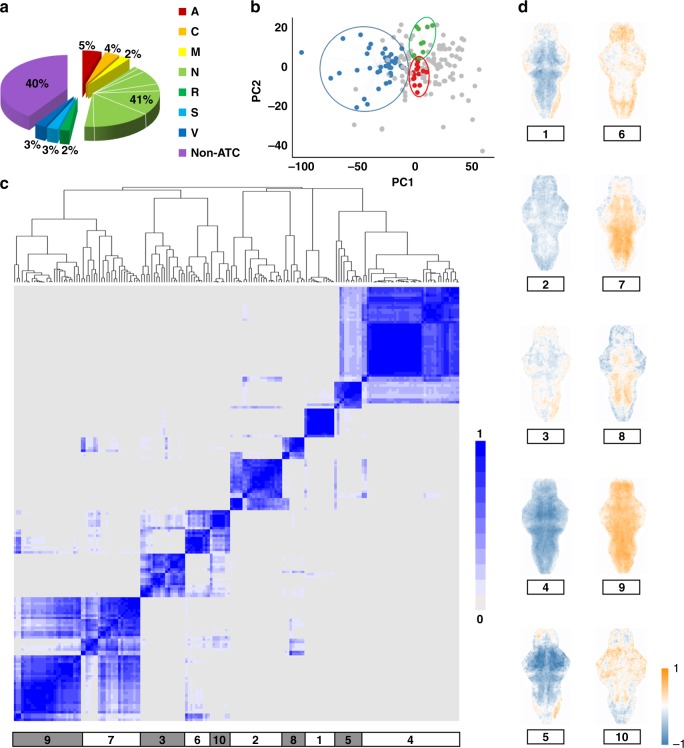
Consensus clustering reveals functional diversity of the drugs. **a** The distribution of the drug library containing clinically used drugs (with ATC codes) and nonclinical compounds. The abbreviations for different ATC categories are: A alimentary tract and metabolism, C cardiovascular system, M musculo-skeletal system, N nervous system, R respiratory system, S sensory organs, V various other drugs. Non-ATC indicates non-clinical compounds without any ATC codes. **b** Visualization of the coherence for the 179 drugs in a two-dimensional space constructed by the first two principal components (PCs). Three clusters of the BAMs were indicated by the blue (cluster 4), green (cluster 8), and red (cluster 3) circles. **c** Consensus clustering identified ten phenotypic BAM clusters. The heat map was the consensus matrix illustrating the frequency of the scenario that two compounds in a pair were clustered together. The color of the heat map is proportional to the frequency scores of the consensus matrix, ranging from 0 to 1. **d** The representative cluster patterns derived by taking mean of all T-score BAMs for drugs in the ten BAM clusters identified

Assuming the BAMs provide a functional description of the therapeutic potential of CNS drugs, we hypothesized that similar BAMs would have an association with a functional drug category that covers a set of compounds, regardless of their chemical structures or molecular mechanisms. To test this hypothesis, we used the complete set of T-score BAMs from the 179 clinical drugs spanning a total of seven different functional categories defined by the WHO ATC system (Fig. 2a; Supplementary Data 1). Principal component analysis (PCA) was then applied to the data to identify the characteristic features of the BAM patterns. Once these characteristic patterns were extracted from all the T-score BAMs, the PCs were used to define a multidimensional vector space, in which each T-score BAM was projected onto the PC vector space by assuming a linear combination of all PCs. In this screen, the top 20 PCs that accounted for the major pattern variation (>50%) across all T-score BAMs were identified, and subsequently used to reconstruct the T-score BAMs while minimizing background noise (Supplementary Fig. 6). After decomposition into the PCs, each characteristic T-score BAM was converted to a dimensionality-reduced Pheno-Print represented by a 20-dimensional vector (Supplementary Fig. 7). We observed that some functionally related drugs shared similar Pheno-Prints. For example, levosulpiride and tiapride, two D2 dopamine receptor antagonists with similar chemical structures that are both used clinically to treat psychiatric disorders, had similar Pheno-Prints (Supplementary Fig. 8). In addition, their Pheno-Prints are similar to that of prochlorperazine, another D2 dopamine receptor antagonist antipsychotic drug with a very different chemical structure than either levosulpiride or tiapride.

We further employed unsupervised classification to detect, in blinded fashion, the intrinsic coherence among similar T-score BAMs, subsequently defined as functional clusters of the tested drugs. Specifically, consensus clustering^21^ (Supplementary Fig. 9) was applied to the library of Pheno-Prints (in 20 dimensions) corresponding to the 179 clinical drugs. As visualized in a two-dimensional space constructed by the first two PCs, some major BAM clusters (e.g. clusters 3, 4, and 8) were already observable (Fig. 2b). Pheno-Print clustering led to the identification of ten BAM clusters (Fig. 2c, Supplementary Fig. 10). For each cluster, the T-score maps for in-cluster drugs were averaged to derive a representative cluster pattern (Fig. 2d). We observed that, while the BAM clusters were relatively distant from each other, there was substantial coherence within each individual BAM cluster for in-cluster drugs. We performed a one-tailed Wilcoxon signed-rank test for each drug, which showed that the Pheno-Prints of within-cluster drugs are significantly (FDR-adjusted *P* < 10^−5^ for all drugs) different than the Pheno-Prints of out-of-cluster drugs. For any drug in a particular cluster, its averaged in-cluster consensus score (the mean of the consensus scores by pairing the drug with every other drug in the same cluster) is significantly higher (FDR-adjusted *P* < 10^−5^ for all drugs) than the averaged out-of-cluster consensus score (the mean of the consensus scores by pairing the drug with every drug outside the cluster), suggesting that the common features of BAMs could be related to specific effects on CNS physiology shared among those in-cluster drugs.

### Functional association with ATC categories

**Fig. 3:**
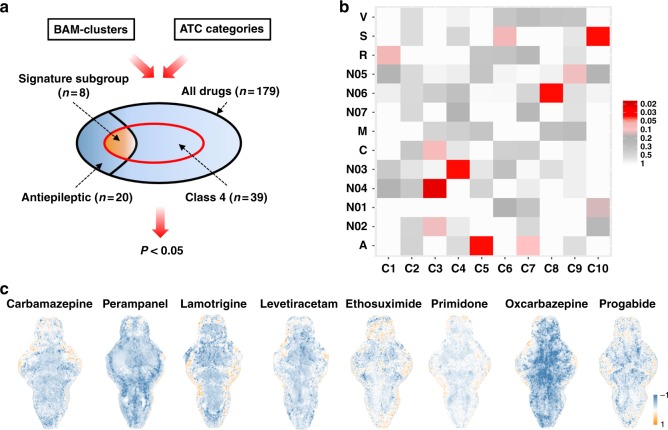
Association of BAM clusters with functional drug categories. **a** Schematic of the hypergeometric test for overrepresentation of a specific Anatomical Therapeutic Chemical (ATC) category by a phenotypic BAM cluster. **b** A heat map illustrating statistical associations between ATC categories and identified BAM cluster. Color coding is based on *p* values derived from the hypergeometric tests (red: lower *p* values or more significant association; gray: higher *p* values or less significant association). **c** The T-score BAMs of the drugs in the signature subgroup of cluster 4, which is significantly associated with N03:Antiepileptics ATC category

**Fig. 4:**
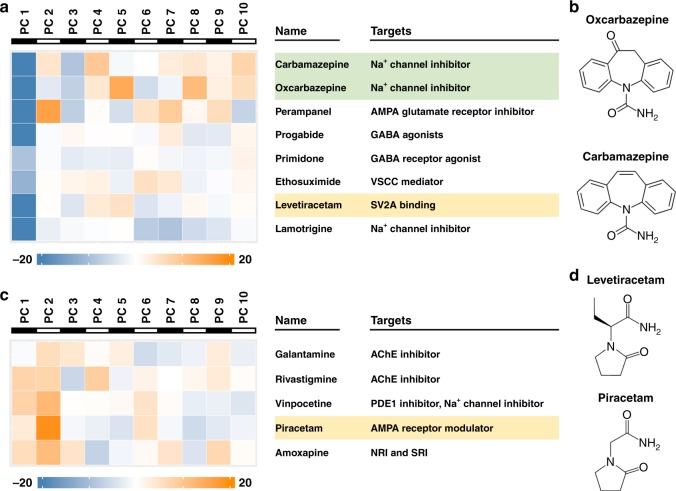
Functional classification of CNS drugs with diverse mechanisms of actions. **a** Molecular targets of the drugs in the signature subgroup of BAM cluster 4 (associated with N03:Antiepileptics ATC category). **b** Similar chemical structures of carbamazepine and oxcarbazepine, green shaded in (**a**), two known antiepileptic drugs. **c** Molecular targets of the drugs in the signature subgroup of BAM cluster 8 (associated with N04:Anti-Parkinson ATC category). **d** Chemical structures of levetiracetam (N03:Antiepileptics) and Piracetam (N06:Psychoanaleptics), yellow shaded in (**a**) and (**c**), which are in distinct ATC categories and find largely different clinical uses despite their structural similarity. Ten principal components (PCs) were listed in panels (**a**) and (**c**)

Next, we sought to identify the relationship between the BAM clusters and the therapeutic function of the 179 compounds in the library, with the clinical use(s) of the compounds classified by the WHO ATC system. To avoid overrepresentation of ATC categories by the identified BAM clusters, a hypergeometric test was performed for statistical analysis of the link between the two systems, which uses a discrete probability distribution to calculate the statistical significance of the subgroup overlaps and is an established method to determine whether a sub-population (e.g., drugs belonging to an ATC category) is overrepresented in a sample (e.g., drugs of a BAM cluster) (Fig. 3a). Interestingly, several BAM clusters (e.g. clusters 3, 4, and 8) were found to have significant overlap with different ATC categories (e.g. N04:Anti-Parkinson Drugs, N03:Antiepileptics, and N06:Psychoanaleptics) (Fig. 3b, *p* < 0.05, hypergeometric tests). In particular, BAM cluster 4 was found to have a strong association with the N03:Antiepileptics ATC category, with a signature subgroup of eight drugs within the cluster that resulted in inhibition of activity across almost the entire brain. This signature subgroup is comprised of carbamazepine, oxcarbazepine, perampanel, lamotrigine, levetiracetam, ethosuximide, primidone, and progabide (Fig. 3c). Within this signature subgroup that clustered together, the structurally similar compounds are thought to exert their clinical effects through binding to a common molecular target, such as the structurally related sodium channel blockers carbamazepine and oxcarbazepine (Fig. 4a, b). More compelling, however, was the observation that compounds with significant diversity in their chemical structure and molecular mechanism also appeared in this signature subgroup (Fig. 4a). Notably, some compounds (e.g. levetiracetam and piracetam) with similar chemical structures, but different ATC-category-based therapeutic functions, were successfully differentiated by their distinct BAMs. Despite their similar chemical structures, levetiracetam and piracetam are employed in different clinical applications; levetiracetam (N03AX14) is a broad-spectrum antiepileptic drug that affects neuronal signaling by binding to and affecting synaptic vesicle glycoprotein 2A (SV2A) function, whereas piracetam (N06BX03) is an allosteric modulator of the AMPA receptor and has been recognized for putative effects as a cognitive enhancer and an adjunctive agent for myoclonus in case reports^22^ (Fig. 4c, d). Our BAM-based clustering successfully separated these two drugs into distinct BAM clusters (clusters 4 and 8) associated with the different ATC categories of N03:Anti-epileptics and N06: Psychoanaleptics, respectively.

### Functional prediction of nonclinically used compounds

The computationally identified association of BAM clusters with clinical ATC categories reveals a drug-screening strategy that is based purely on brain physiology, which does not require any prior knowledge of chemical structure or molecular target. For an unknown compound, the relegation of its functional activity map to an ATC-associated BAM cluster would indicate higher probability for the compound to be a hit for that particular ATC category. We sought to further validate our BAM cluster method by attempting to predict the neuropharmacology of an additional test set comprised of 121 compounds currently without ATC codes (Supplementary Data 2). This test set was constructed in a manner to ensure sufficiently large coverage of different molecular targets (Supplementary Fig. 11). The goal during this portion was to employ BAM-based phenotyping to predict the potential therapeutic role of these nonclinical compounds using a two-step prediction methodology. First, the clustering result from the training set (179 ATC-coded drugs; Supplementary Data 1) was used to generate a random forest classifier (Fig. 2c), which is a machine learning paradigm that employs an ensemble of decision trees for robust classifications. This method computationally assigned the 121 compounds into one of the ten BAM clusters (a–j) previously generated from the training set (Supplementary Fig. 12). Second, compounds from the test set that were assigned to those clusters with significant association with ATC categories (clusters 3, 4, and 8) were further prioritized based on the Pearson correlation coefficient (therapeutic potential) between the compound’s Pheno-Print and the centroid (in 20-dimensional PC space) of all drugs in the signature subgroup of a particular BAM cluster (Supplementary Fig. 13). This correlation was then used as a quantitative index to rank the therapeutic potential of the classified compounds.

**Fig. 5:**
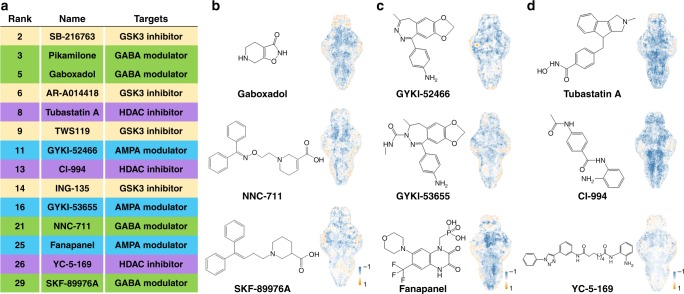
Prediction of the nonclinical compounds using the HT-BAMing technology. **a** List of top-rated compounds predicted to be potent N03:Antiepileptic drug candidates. Compounds sharing the same molecular targets were shaded in the same color. **b−d** The chemical structures of several predicted potent antiepileptic compounds with distinct molecular targets, including **b** GABA modulators (gaboxadol, NNC-711 and SKF-89976), **c** AMPA modulators (GYKI-52466, GYKI-53655 and fanapanel), and **d** HDAC inhibitors (tubastatin A, CI-994 and YC-5-169)

In parsing the therapeutic potential of compounds, we noted that predicted cluster 4 was enriched for potential antiepileptics among the top-ranked compounds: of the top 30 compounds, there is literature support for antiepileptic properties for more than 47% (Fig. 5a, Supplementary Table 1). In particular, gaboxadol, SKF89976A, and NNC-711 have previously been reported to have antiepileptic activity in animal models^23–25^. SKF89976A and NNC-711 share highly similar chemical structures (Fig. 5b) and are both inhibitors of GABA uptake. Another group of N03:Antiepileptics candidates were AMPA receptor antagonists, including GYKI-52466, GYKI-53655, and fanapanel (Fig. 5a, c), whose antiepileptics properties are supported by the literature^26,27^. Of note, AMPA receptor antagonists are a new addition to the antiepileptic armamentarium, as evidenced by the recent approval of the first anti-seizure drug in this class, perampanel, a selective, noncompetitive antagonist used as adjunctive therapy in partial-onset seizures and the treatment of primary generalized tonic-clonic seizures^28^.

In addition to compound classes with previously reported antiepileptic activity, we noted that the compound CI-994 was also predicted as a top-ranked N03:Antiepileptics candidate (Fig. 5a, d). CI-994 is a known modulator of epigenetic mechanisms through its activity as a sub-class I selective histone deacetylase (HDAC) inhibitor^29,30^. Structurally, CI-994 is an acetylated derivative of the substituted benzamide dinaline, which was originally discovered as a CNS-penetrant, anticonvulsant agent based upon in vivo testing in rodents^31,32^. Consistent with a role for HDAC inhibition as being a factor in driving the BAM profiles that cluster CI-994 within the predicted N03:Anti-epileptics group, an additional top-ranked prediction, YC-5-169, shares key structural feature with CI-994, namely an *ortho*-aminoanilide group that chelates zinc atoms in the active site of HDACs. Furthermore, a second compound, tubastatin-A, was also among the top-ranked predicted compounds in the N03:Antiepileptics group; due to its hydroxamic acid rather than *ortho*-aminoanilide group, tubastatin A has a broader activity profile that includes HDAC6 and HDAC10 inhibition in addition to class I HDACs at higher doses^33^. These results suggest that inhibition of HDACs may present a novel mechanism of action for development of future anticonvulsants (Fig. 5d). To test the reliability of this HT-BAMing-based compound screening strategy, we repeated the analysis on a small subgroup of 20 compounds randomly selected from the test set. As shown in Supplementary Fig. 14, most of the T-score BAMs were similar to that acquired in the first trial. After inputting the re-acquired BAMs into the classifier, we found that 90% (18 of 20) of the compounds were assigned to the same cluster as in the first trial, indicating our screening strategy and analysis is highly reproducible. In another validation experiment, we tested seven additional clinical antiepileptics that were not present in either the training or test sets. Using only their BAMs as inputs, five of the seven drugs (~70%) were classified to cluster 4 (N03:Antiepileptics cluster) (Supplementary Fig. 15), and were ranked among the top hits within the cluster when added to the results from the test set (Supplementary Table 2). This included the inhibitory neurotransmitter GABA, the GABA(A) receptor-positive allosteric modulator and possible lactate dehydrogenase inhibitor stiripentol, the voltage-sensitive calcium channel antagonists methsuximide and trimethadione, and the sodium channel antagonist phenytoin. Importantly, these results demonstrate the generalizability of our findings to compounds not tested in the original screen and that we anticipate that as we continue to add compounds to our training set library our classification will improve.

### Validation of the machine learning predictions

**Fig. 6:**
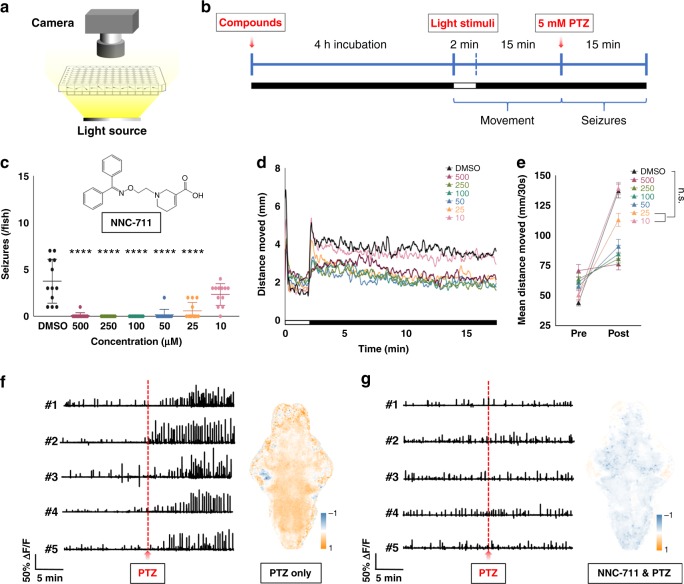
Validation of the hit compounds using behavioral test in PTZ-seizure animal model. **a** Schematic of the setup for the behavioral test. **b** The experimental protocol. **c** The seizure count over 15 min in larvae exposed to 5 mM PTZ after 4-h preincubation with NNC-711 at different concentrations (10 ~ 500 μM). A dose-dependent reduction in seizures is noted. Error bars indicate standard error of the mean (s.e.m.), *n* = 12, **** indicates *p* < 0.0001 by one-way ANOVA tests. **d** Representative results for the movement behavioral responses with NNC-711 preincubation at different concentrations (10 ~ 500 μM). **e** The statistical analysis of the locomotive behavior of the larvae in response to the preincubation with NNC-711 at different concentrations (10 ~ 500 μM). Each larva’s movement was monitored for 15 min after a 2 min light stimulation. Error bars indicate s.e.m., *n* = 12. Larvae treated with DMSO were used as a control, “n.s.” stands for no significant difference with *p* > 0.05 by ANOVA test. **f** Traces of calcium fluctuation from five larval brains and the associated T-score BAM showing the seizure activity induced by 5 mM PTZ treatment. **g** Traces of calcium fluctuation from five larval brain and the associated T-score BAM showing the reduction of seizure activity in PTZ-larvae by a 4 h treatment of NNC-711 at 10 µM

To validate the results of our machine learning-based therapeutic classification of activities of bioactive compounds, we selected the 14 top-ranked antiepileptic candidates (Supplementary Table 3) for testing in the pentylenetetrazole (PTZ)-induced zebrafish seizure model (Supplementary Movie 1). PTZ is a noncompetitive antagonist of GABA(A) receptors that has been widely used to study seizure phenomenon across a wide range of vertebrates, including zebrafish^34,35^. By blocking inhibitory neurotransmission mediated by GABA(A) receptors, this compound leads to a proconvulsant effect. In these experiments, following a 4-h drug incubation period, zebrafish larvae were premonitored for normal movements/swimming behavior for 17 min. At 2 min, the lights were turned off, which should normally induce a rapid increase in movement; failure to increase movement suggested fish were sedated or otherwise unable to respond normally to the stimulus. At the 17-min mark, the larvae were treated with PTZ to a final concentration of 5 mM, and the number of seizures per larva and the average number of seizures at each concentration were quantified for 15 min following PTZ exposure (Fig. 6a, b, and Supplementary Movie 2). After excluding two compounds insoluble at higher concentrations, larvae pretreated with seven of the 14 compounds tested were found to have fewer seizures than the DMSO-treated control larvae without sedating the fish (Supplementary Table 3). For example, pretreatment with NNC-711 resulted in marked reduction in seizures following PTZ treatment. Although partial sedation was observed at higher concentrations, at the lower dose of 25 μM, NNC-711 reduced the number of seizures without any apparent effect on zebrafish locomotive behavior (Fig. 6c, d). Such pharmacological effects could also be observed in the diametrically opposed BAMs of zebrafish treated in the same manner (Fig. 6e−g). NNC-711 is thought to principally act through the inhibition of GABA uptake from the synaptic cleft and glia via inhibition of SLC6A1 (solute carrier family 6 member 1; (GAT1)) leading to increased GABA-ergic neurotransmission. These observations in the PTZ zebrafish seizure model are therefore consistent with the reported anticonvulsant efficacy of NNC-711 in rat models of induced epilepsy^36–38^. Another candidate predicted from the BAMs analysis to have antiepileptic activity was GYKI-52466, a noncompetitive AMPA receptor antagonist, which was tested and confirmed in our PTZ-seizure model (Supplementary Fig. 16a). In agreement, GYKI-52466 has been shown previously to have anticonvulsant activity in a kainic-acid-induced seizure model in mice^39^.

In addition to the examples of NNC-711 and GYKI-52466, for which retrospective support can be provided for their efficacy from the literature, there were also several nonobvious activities of the predicted hits when tested in the PTZ model (Supplementary Fig. 16). For example, volinanserin, a highly selective 5-HT2A receptor antagonist that has been tested as a potential antipsychotic, antidepressant, and sleep aid in preclinical models^40–42^, but has not been reported to have anti-seizure activity, reduced seizures in our PTZ-seizure model at relatively low does (Supplementary Fig. 16c). Yangonin is a kavalactone that, in the oncology literature, induces autophagy and inhibits the mTOR pathway, and is a novel CB1 receptor ligand, but with no reported anti-seizure activity^43,44^ (Supplementary Fig. 16d). Also, the HDAC6 inhibitor tubastatin-A reduced seizure count at a final concentration of 500 μM, the upper end of the concentration range employed in these studies (Supplementary Fig. 16e).

Overall, the hit compounds employed in the behavior validation studies were selected based on their Pearson correlation coefficient rank within the N03:Anti-epileptic cluster (all had a correlation coefficient of 0.89–0.99), and with an eye towards including a diverse array of putative mechanisms of action, rather than based on chemical structure or previously published results on their clinical effects. That seven of the 14 compounds tested demonstrated anti-seizure activity in the PTZ model supports the HT-BAMing technology using zebrafish as a physiology-based screening tool for pharmacological discovery.

## Discussion

With the advent of recombinant DNA technology and tissue culture techniques, the early stages of pharmaceutical discovery for CNS diseases have increasingly relied on simplified targets consisting of recombinant proteins or heterologous cellular models^4^. More recently, screening methods using organism models have been developed^6,7,10^, but these platforms are limited in their ability to perform large-scale chemical screens based on direct evaluation of organ-specific physiology in a complex animal model. Here, we describe a strategy for functional CNS drug screening in larval zebrafish by using HT-BAMing technology. This technique is capable of rapidly assessing changes in brain physiology and activity in response to exposure to a compound at the level of cellular resolution across an entire zebrafish brain. While we here only differentiate 2D brain regions in a z-projected image, and used the associated BAM information for computational analysis, as an extension of this screening paradigm, 3D brain structural information can be included for analysis with the help of more advanced imaging technique.

Using HT-BAMing, we generated a collection of BAMs, each of which reflects the changes in brain physiology caused by exposure to a particular compound in a library of bioactive compounds and approved drugs. As part of this screening strategy, we implemented a computational and machine learning-based approach to analyze the large-scale BAM dataset, and demonstrated successful prediction of drug leads for neurological diseases without any prior chemical or molecular knowledge of the compound library. Double-blind analysis of a training set containing 179 CNS-active drugs revealed that the phenotypic BAMs naturally form coherent clusters, which were further discovered to have strong association with the clinical usage of those medications, based on their functional WHO ATC classification. This strategy was then validated in a test set of 121 nonclinical compounds without an ATC code. By employing the coherent BAM clusters derived from the set of 179 ATC-coded drugs as a classifier, we made several interesting predictions about the potential therapeutic application of these compounds. This association between BAM clusters and ATC categories bridges the enormous gap between high-throughput physiology phenotyping and the potential therapeutic applications of unknown compounds.

Notably, the BAM-based clustering and prediction are solely based on the modulation of brain activity following exposure to a compound. Therefore, it is not surprising to see a dramatic diversity, both in chemical structures and molecular targets, for compounds within the identified BAM clusters. In particular, for BAM cluster 4, which is strongly associated with the N03:Anti-epileptics ATC category, the signature subgroup for this cluster contains drugs targeting six different major molecular targets. This result suggests that our BAM-based assay provides a drug-screening platform with the potential to accommodate the pathophysiologically complex nature of many brain disorders, including epilepsy and other disorders with network imbalance such as those caused by neurodegeneration^45^^,^ or autism spectrum disorders such as Rett syndrome^46^ and Pitt-Hopkins syndrome^47^. Indeed, screening of the test set of 121 compounds correctly clustered potent N03:Anti-epileptic compounds, and, critically, identified several potential lead structures for development of antiepileptics, including volinanserin and yangonin.

In our efforts to validate the prediction results using the PTZ-seizure model, seven of the 14 compounds tested (50%) decreased seizure frequency even at concentrations where they did not significantly impact the larvae locomotive activity. Given that PTZ is a noncompetitive GABA(A) antagonist, the fact that the other seven compounds tested were negative in the PTZ model may reflect limited sensitivity of the PTZ model to detect anti-seizure activity mediated through a pathway other than GABA rather than true lack of efficacy as potential anti-seizure agents^48^.

This exemplifies the power of the HT-BAMing-based screening strategy to function independent of any particular pharmacological model, and also highlights the necessity of secondary functional phenotyping methods that are not limited by our knowledge of the pathophysiology of a disorder at the molecular level or to only a limited number of pharmacological models that may have inherent biases in their sensitivity. This is particularly powerful in a disorder such as epilepsy, in which seizures represent the common phenotypic expression of a multifactorial propensity towards seizure activity. Identification of compounds with previously unrecognized antiepileptic activity may improve our understanding of changes at the genetic, epigenetic and cellular levels that create and propagate a chronic tendency towards seizure.

Although we focused on the analysis of anti-seizure drugs in this proof-of-concept study, our results suggest that the HT-BAMing-based screening strategy can be applied to advance pharmacological discovery in other complex brain diseases. Two other BAM clusters from the training set, clusters 3 and 8, had significant overlap with ATC categories N04:Anti-Parkinson Drugs and N06:Psychoanaleptics, respectively. The top-ranked prediction from the N04:Anti-Parkinson Drugs ATC category, berberine, has been shown in mice to increase dopamine levels, similar to the pro-dopaminergic drugs entacopone and ropinirole^49^. Also, the top hit in the N06:Psychoanaleptics category, hydroxytacrine, shares in common the mechanism of acetylcholinesterase inhibition that the Alzheimer’s disease drugs galantamine and rivastigmine in the BAM cluster 8 have. It is expected that the use of ATC clinical drugs to construct a larger training set should facilitate the translation of the pharmacological profiles of non-ATC compounds from zebrafish to humans^50^.

Use of HT-BAMing technology has the potential to provide insight into mechanisms of action of poorly understood pharmacological agents and novel compounds, particularly as the number of reference BAMs obtained from treatment with well-characterized compounds with known mechanisms of action increases. The HT-BAMing-based screening strategy presented here can be refined in several ways. For example, the clustering analysis can be refined by taking advantage of more advanced machine learning methods such as recursive cortical network^51^ or deep learning algorithms^52^; relevant structure-specific information in the BAMs may be spatially encoded for analysis. The proof-of-concept study presented here tested each compound at a single dose by introducing the compound into the environment of the zebrafish at 10 μM concentration; one limitation of this design and in the interpretation of data is the fact that it is unclear how this concentration corresponds to the concentration achieved in the larval CNS, and moreover how this 10 μM concentration relates to typical brain exposure levels achieved in humans. In addition, the binding specificity of compounds may change with changes in concentration, and assessing changes in the BAM across a variety of concentrations may elucidate new pharmacological effects of clinical compounds or predict off-target effects in novel or investigative compounds. Obtaining BAMs at several time-points following one-time or repeated dosing may help identify compounds capable of affecting the course of CNS disorders through longer-term changes in CNS activity. Finally, more advanced whole-brain imaging and analysis methods, including registration to brain atlases^53^ could help identify specific cell populations affected by compounds. Future work using the BAM screening technology presented here will address these concerns, with an eye towards illuminating the underlying pathophysiology of complex disorders of the CNS. Finally, the use of HT-BAMing in conjunction with zebrafish models of CNS disorders created through the introduction of causal genetic variants using CRISPR/Cas and related genome engineering strategies has tremendous potential to advance an understanding of systems neuropharmacology and to assist in the discovery of novel disease-modifying pharmacological agents to expand the treatment options available to patients with CNS disorders.

## Methods

### Zebrafish line

The transgenic zebrafish line *elavl3*:GCaMP5G was acquired from Dr. Michael Orger, and was maintained in aquaria under standard laboratory conditions (at 28 °C under a cycle of 14 h light, 10 h dark). Larvae of 6–8 dpf were used in the HT-BAMing experiments. All animal work was carried out with prior approval from the animal ethical committee of City University of Hong Kong and was in accordance with local animal care guidelines.

### Microfluidic chip

A negative mold of the chip design was fabricated by high-resolution (30 μm resolution) computer numeric control machining using a plain copper plate. The transparent flow channels were then made by molding from the copper molds using polydimethylsiloxane (PDMS). After curing for 12 h, the PDMS structures were released from the molds and then bonded to glass substrate after plasma treatment to form the final microfluidic chip.

### Microfluidic handling of larval zebrafish

To enhance the larvae processing throughput, we developed a larvae loading and transportation system to enable automatic feeding of larvae into the microfluidic chip. The automated system was based on digitally controlled fluidic circuitry using syringe pumps, electromagnetic valves, and video detection technique (Supplementary Fig. 1a). Two syringe pumps (RSP01-B; RISTRON) were used to load the zebrafish larvae from a reservoir into the capillary fluidic circuitry. A NIDAQ input−output card (NI USB 6525) was used to digitally control the pumps and the electromagnetic fluidic valves (WK04-010-0.5/1-NC; Wokun Technology) to perform automated control of larva handling cycles. A video detection module was developed to detect the passage and the larva head direction, which was used as a trigger signal to the direction-switching-loop module (Supplementary Fig. 1b). This module was designed to adjust the direction of the larva after loading from the reservoir to ensure each larva was in a tail-forward direction before being loaded into the microfluidic chip. For fast larva detection, an algorithm was developed in-house to extract every frame from the real-time recording and convert it to a binary image, and the head portion was then identified by simple center of gravity detection (Supplementary Fig. 1c).

### Compound library and chemical treatment

We screened a library of structurally and target-diverse compounds, including a training set comprised of 179 clinically used CNS drugs spanning seven different WHO ATC system classes, and 121 nonclinical compounds as a test set. All compounds were dissolved in DMSO as a vehicle as ~10 mM stock solutions. Treatment of the larvae was performed simply by switching the perfusion solution after immobilizing larvae in the microfluidic chip (Supplementary Fig. 1d). Final concentrations of compounds tested in the primary screen were ~10 μM.

### Microscopy

Imaging was performed on a fully automated inverted fluorescent microscope (Olympus IX81) equipped with a cooled sCMOS camera (Neo, ANDOR) with a ×10  (NA, 0.4) objective. Micro-manager 1.4 was installed to control the microscope. All the screening data were acquired using epifluorescence microscopy, the calcium transients were recorded at two planes (the top plane and the middle plane) separated by 100 μm distance in each larva. For high resolution, confocal imaging and Leica SP8 microscope with a resonant scanner was used. Confocal microscopy was only used to demonstrate the stability of the fish trapping strategy for the immobilization of larvae without causing any disturbance for high-resolution imaging, which is shown in Fig. 1d. For that particular image, a total height of 200 μm was scanned with 25 sections (8 μm *z*-resolution).

### BAM generation from calcium imaging data

The procedures for analyzing brain-wide activity were illustrated in Fig. 1a and Supplementary Fig. 2. Briefly, a heat map was used to intuitively display the activation level of the brain. To map brain activity, every collected frame from calcium imaging was first meshed into small regions of interest (ROIs), each with a size of 15.21 µm^2^. The time series of fluorescence signal in each ROI was then filtered by a high pass filter with a cut-off frequency of 0.2 Hz to remove the interference from illumination fluctuations. The resulting trace was used to calculate and visualize calcium transients as a measure of neural activity. For analyzing the calcium transients, a threshold was applied to detect the change in calcium levels defined by the mean plus two times the standard deviation of the Δ*F*/*F* curve derived from the recording of calcium fluorescence.

For each compound, we obtained the count of calcium transients from five independent zebrafish larvae over a 10-min period before and a 15-min period after compound treatment. Before comparison across different samples, all the images were first resized and aligned to a uniform zebrafish brain template with the following process: for each raw image, the dark background was first removed to extract the fluorescent brain region, which was then mapped to the standard template via specific transformative adjustments (e.g. rotation, translation) using the brain center-line as a registration landmark and such that the symmetry with respect to the center-line was maximized in the resulted image. Lastly, the fish eye region was further removed by taking the regions within the template for downstream analysis (Supplementary Fig. 2). A BAM *A*=[***a***_*ij*_] was then derived by taking the change (increase or decrease) of calcium transient counts in each ROI before (*t*_0_) and after chemical treatment (*t*_1_), and summed across multiple layers along the *Z*-axis:

1 $${\boldsymbol{a}}_{ij} = \mathop {\sum }\limits_k {\boldsymbol{c}}_{ijk}^{t_1}{\boldsymbol{ - c}}_{ijk}^{t_0},$$

where ${\boldsymbol{c}}_{ijk}^{t_0}$ and ${\boldsymbol{c}}_{ijk}^{t_1}$ represent the calcium transient counts of the ROI at row *i* and column *j* of the *k*th layer before and after chemical treatment, respectively.

### T-score BAM calculation

For quantitative assessment of the statistical significance of brain activity regulation by a compound, a matrix of T-scores (T-score BAM),*T* = [*t*_*ij*_] was calculated for each ROIs across the five BAMs from different biological replicates by:

2 $${\boldsymbol{t}}_{ij} = \frac{{\overline {{\boldsymbol{a}}_{ij}} }}{{{\boldsymbol{S}}_{{\boldsymbol{a}}_{ij}}/\sqrt n }},$$

where $\overline {{\boldsymbol{a}}_{ij}}$ was the mean brain activity score of the ROI at row *i* and column *j* of the five BAMs, ${\boldsymbol{s}}_{{\boldsymbol{a}}_{ij}}$was the standard deviation of the mean, and *n* = 5 was the number of biological replicates.

### Identification of phenotypic BAM clusters

Using the T-score BAMs for the training set of 179 clinical drugs, we employed unsupervised classification to dissect the phenotypic diversity. First, to reduce the dimensionality and noise, PCA was applied to all T-score BAMs. The top 20 principal components (PCs) were used to construct the Pheno-Prints for further analysis. Next, we employed consensus clustering based on hierarchical clustering with bootstrap resampling (*n* = 1000)^21^. Ten optimal phenotypic BAM clusters were identified (Fig. 2c), as the area under the empirical cumulative distributions functions curve did not increase substantially (<1%) from 10 to 11 clusters, and so on (Supplementary Fig. 9). For each BAM cluster, a representative cluster pattern was calculated by taking the mean of the T-score BAMs over all in-cluster compounds (Fig. 2d).

### Functional prediction using machine learning

To link identified phenotypic BAM clusters to therapeutic drug categories, we performed hypergeometric tests for overrepresentation (Fig. 3a). In our test for overrepresentation of an ATC category in a BAM cluster, the hypergeometric *p* value is calculated as the probability of observing *k* or more drugs of an ATC category (from the whole population of all 179 drugs in the training set) in total *n* drugs of a specific BAM cluster. For each pair of BAM cluster and ATC category, the resulting *p* values were used to identify nominally statistically significant associations (*p* < 0.05). For ATC-associated BAM clusters, the significant overlap between a BAM cluster and an ATC category was defined as the signature subgroup. To predict the therapeutic function of nonclinical compounds in the test set, a two-step prediction strategy was used. First, a random forest classifier was built using R package “randomForest” (with parameter *ntree* set to 100) based on the clustering results from the training set of 179 clinical drugs, in order to classify the 121 nonclinical compounds into the ten phenotypic BAM clusters. For this purpose, all T-score BAMs of the test set were projected to the PC space of the training set to derive their Pheno-Prints and used as inputs for the classifier. Secondly, for compounds that were relegated to ATC-associated BAM clusters, the prediction was further prioritized based on the Pearson correlation coefficient between each compound’s Pheno-Print and the signature subgroup’s centroid in the PC space.

### Behavioral validation of predicted antiepileptic compounds

The experimental protocol uses a 96-well plate, with one zebrafish larva (5 days post-fertilization) in 90 μL of fish water in each well. 10 μL of a 10× solution of compound in 10% DMSO was added to each well to achieve the final concentration shown in the data, and for a final concentration of 1% DMSO. For each concentration, there were 12 fish, and each plate had a group of control larva treated with 1% DMSO (final concentration). Final concentrations of compounds were 500, 250, and 100 μM, except in the case of compounds found to be highly insoluble in water, in which cases the final concentrations were 50, 25, and 10 μM; insoluble compounds tested at these lower concentrations were 7,8-dihydroxyflavone, AR-A014418, P7C3, volinanserin, and yangonin. The larvae were pretreated with compound for 4 h, followed by a 2-min dark stimulation to ensure fish were not sedated, followed by a 15-min period of observation for deviation from the anticipated normal movement/swimming behavior. A quick change from light to dark induces an increase in activity in zebrafish. After a 15-min observation period, the larvae were treated with PTZ to a final concentration of 5 mM, and the number of seizures per larva at each concentration quantified for 15 min following PTZ treatment. The DanioVision platform (Noldus) was used to perform the animal behavioral recording and analysis.

## Electronic supplementary material

Supplementary Information

Description of Additional Supplementary Files

Supplementary Movie 1

Supplementary Movie 2

Supplementary Data 1

Supplementary Data 2

Reporting Summary

## Data Availability

The primary data that support the findings of this study are available from the corresponding author upon request.
