# Supplementary material for: High-throughput brain activity mapping and machine learning as a foundation for systems neuropharmacology
Source: Nat Commun. 2018 Dec 3;9:5142. doi: 10.1038/s41467-018-07289-5 (PMC6277389; doi:10.1038/s41467-018-07289-5)
Supplement: Supplementary file 1 — Supplementary Information [file 41467_2018_7289_MOESM1_ESM.pdf]

High-throughput Brain Activity Mapping and Machine Learning as a Foundation for Systems Neuropharmacology

Lin *et al*.

SUPPLEMENTARY FIGURES

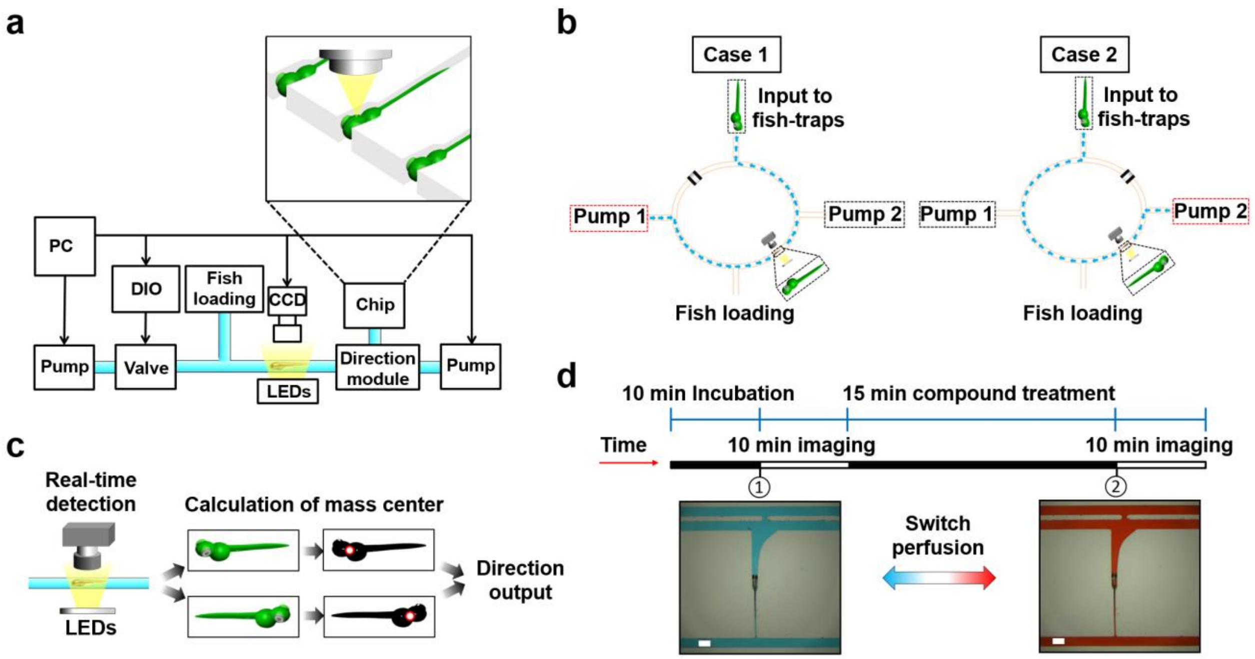

Supplementary Figure 1. Automated system for handling larval zebrafish and drug perfusion. (**a**) Schematic of the automated system for manipulation of zebrafish larvae. (**b**) Illustration of the direction-switching-loop. For a larva loaded with tail facing forward (Case 1), the left pump 1 (in red box) is engaged to push the larva into the Fish-Trap chip. For a larva loaded with head facing forward (Case 2), the right pump (in red box) is engaged to push the larva into the Fish-Trap chip. The flow direction is indicated by the blue dash line. (**c**) The video-detection module coupled with an in-house developed image processing algorithm for differentiating fish head and tail. (**d**) Diagram showing the drug treatment to larvae in a Fish-Trap by switching the perfusion solution. Scale bar, 1 mm.

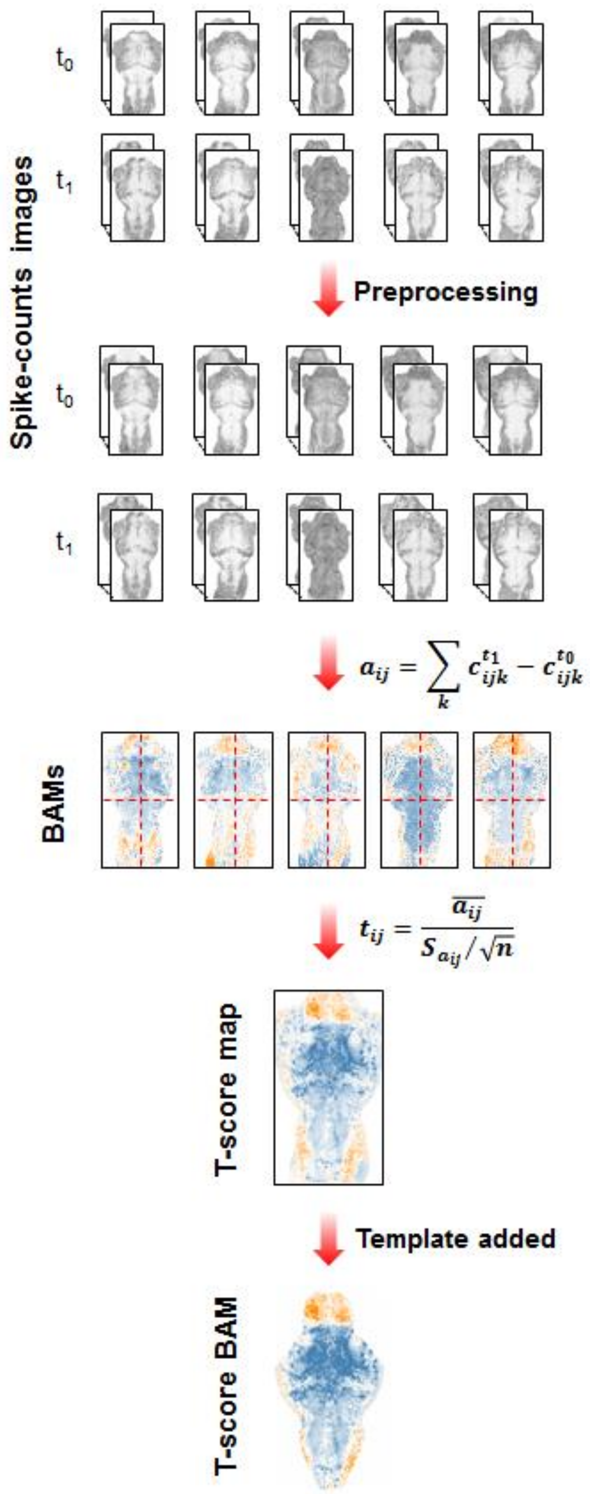

Supplementary Figure 2. Analytical workflow for the generation of T-score brain activity maps (T-score BAMs). The key steps for the calculation of a T-score BAM involve image-based preprocessing of calcium spike counts, generation of brain activity maps, calculation of a T-score map, and fitting with a uniform zebrafish brain template (more details in **Methods**).

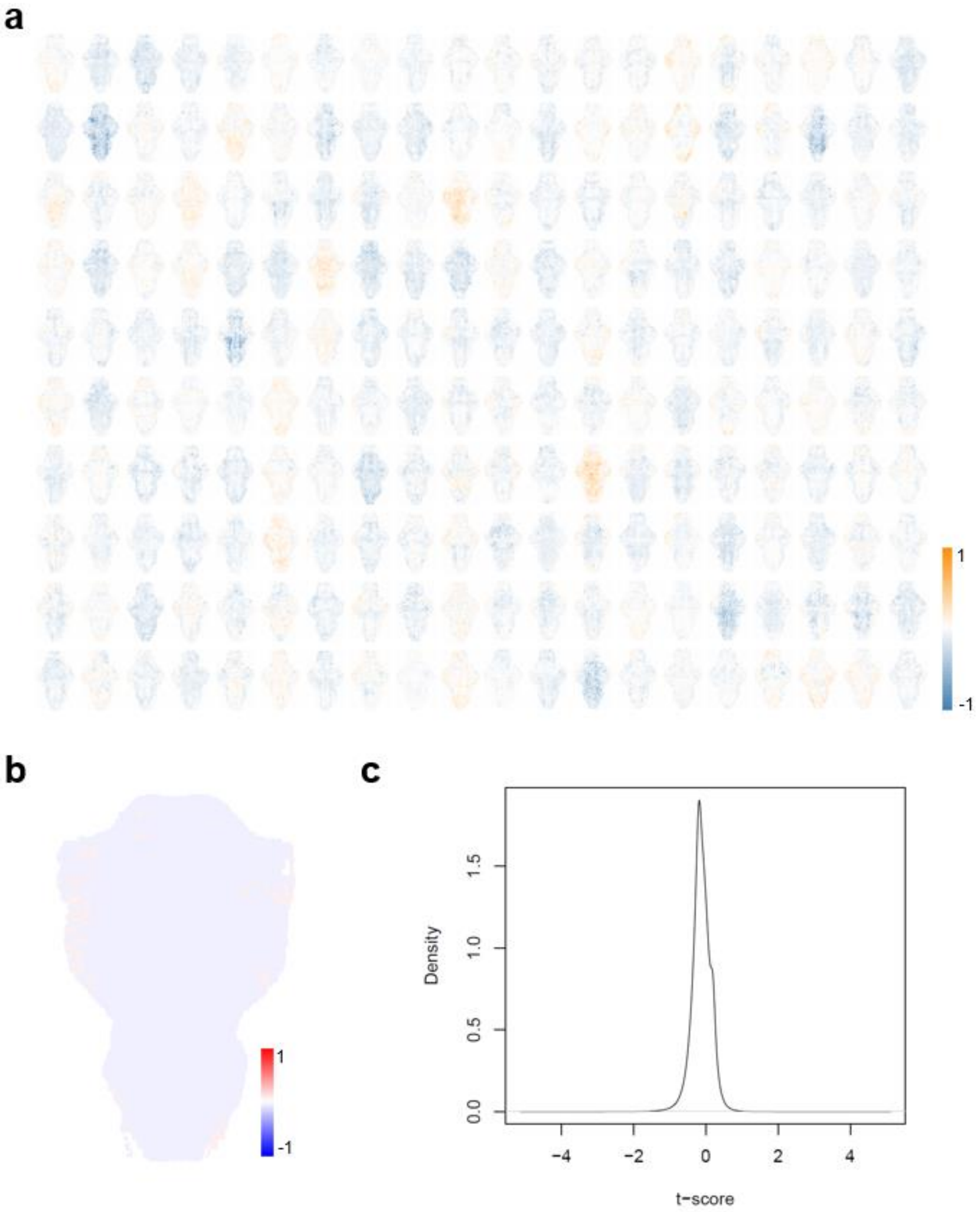

Supplementary Figure 3. Background T-score BAMs calculated from DMSO-treated control groups. From the total 50 DMSO-treated larvae, we randomly selected 5 samples to calculate a background T-score BAM, and this step was repeated for 1000 times. 200 background T-score BAMs were randomly selected and shown in (**a**). The average of all 1000 background T-score BAMs was shown in **(b).** The pooled distribution of the T-scores of all 1000 background T-score BAMs was shown in **(c)**, which were white-noise-like and were distinct from those drug-treated samples.

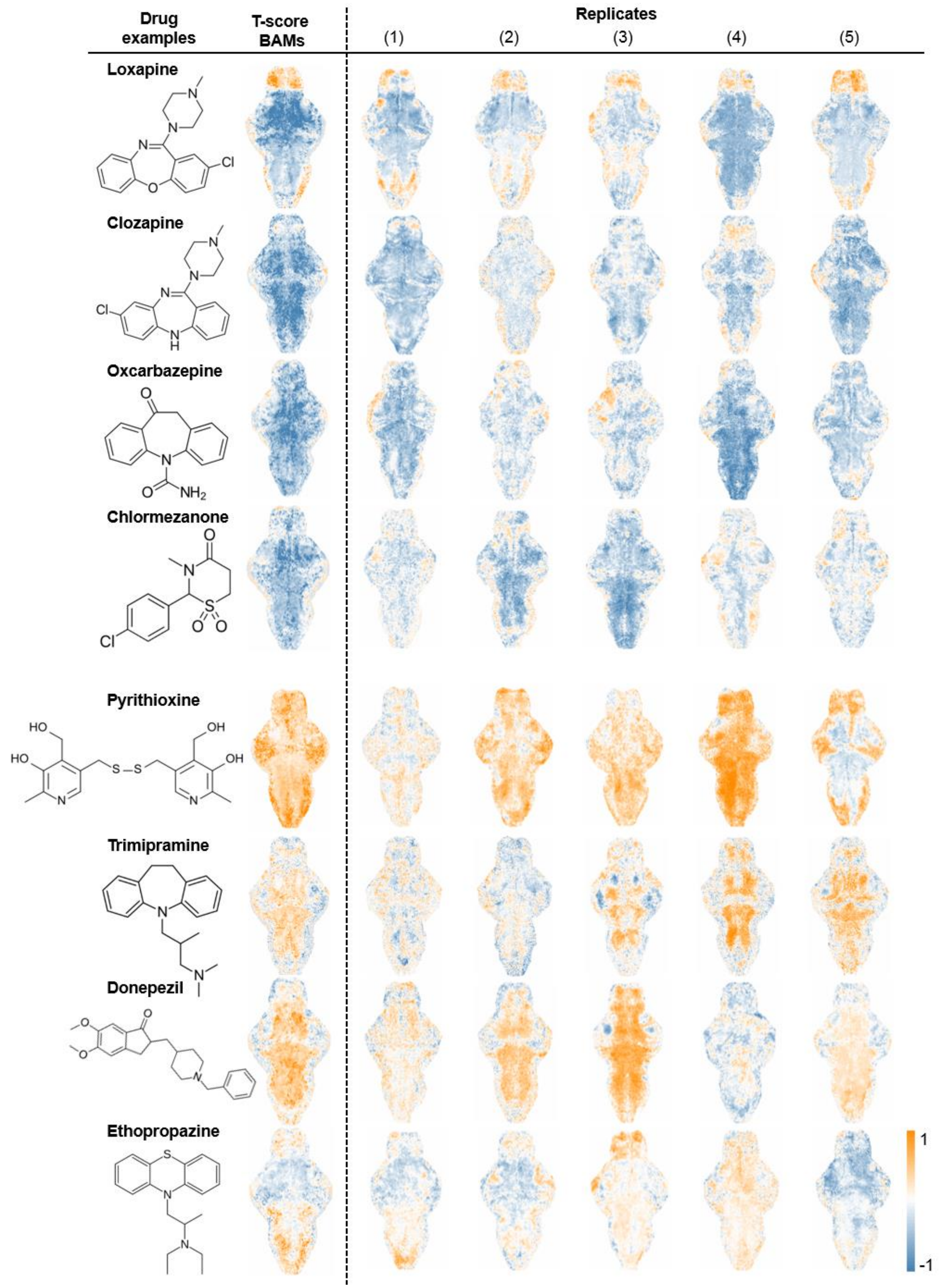

Supplementary Figure 4. Sample drugs of various chemical structures that generated unique T-score BAMs. The images on the left of the dash-line are the composite T-score BAMs derived from the five BAMs from different biological replicates (on the right of the dash-line).

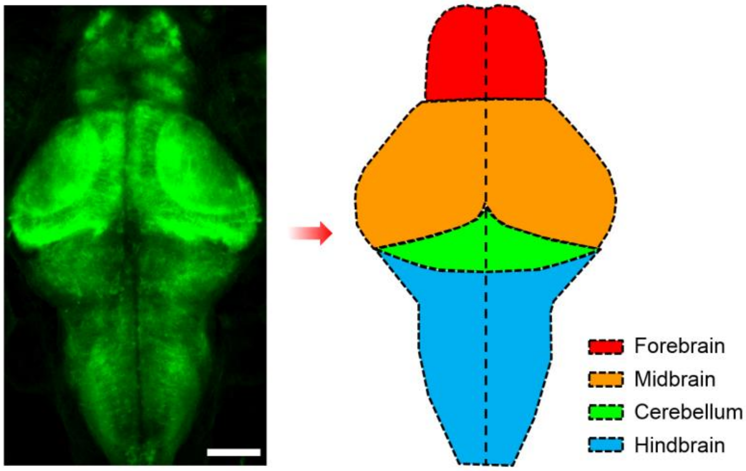

Supplementary Figure 5. Illustration of the brain of a larval zebrafish and the associated brain template used to standardize the BAMs. Major brain regions are outlined by the dash-line and are labelled with different colors. Scale bar, 100μm.

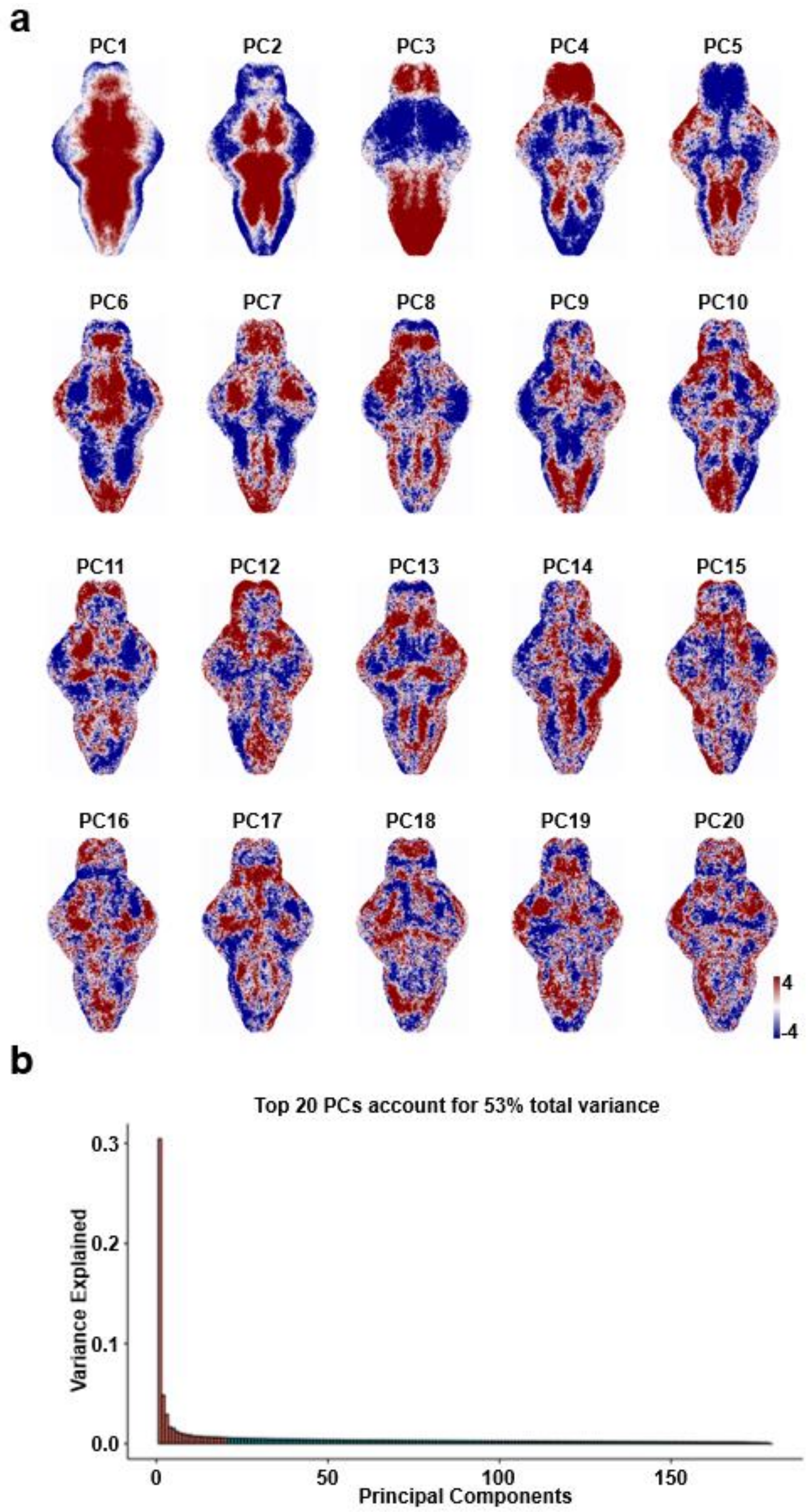

Supplementary Figure 6. The top 20 principal components (PCs) account for the major variation of all the T-score BAMs. (**a**) Images of the pheno-print vectors of the top 20 PCs. (**b**) Quantification of the variance explained by the top 20 PCs (53%).

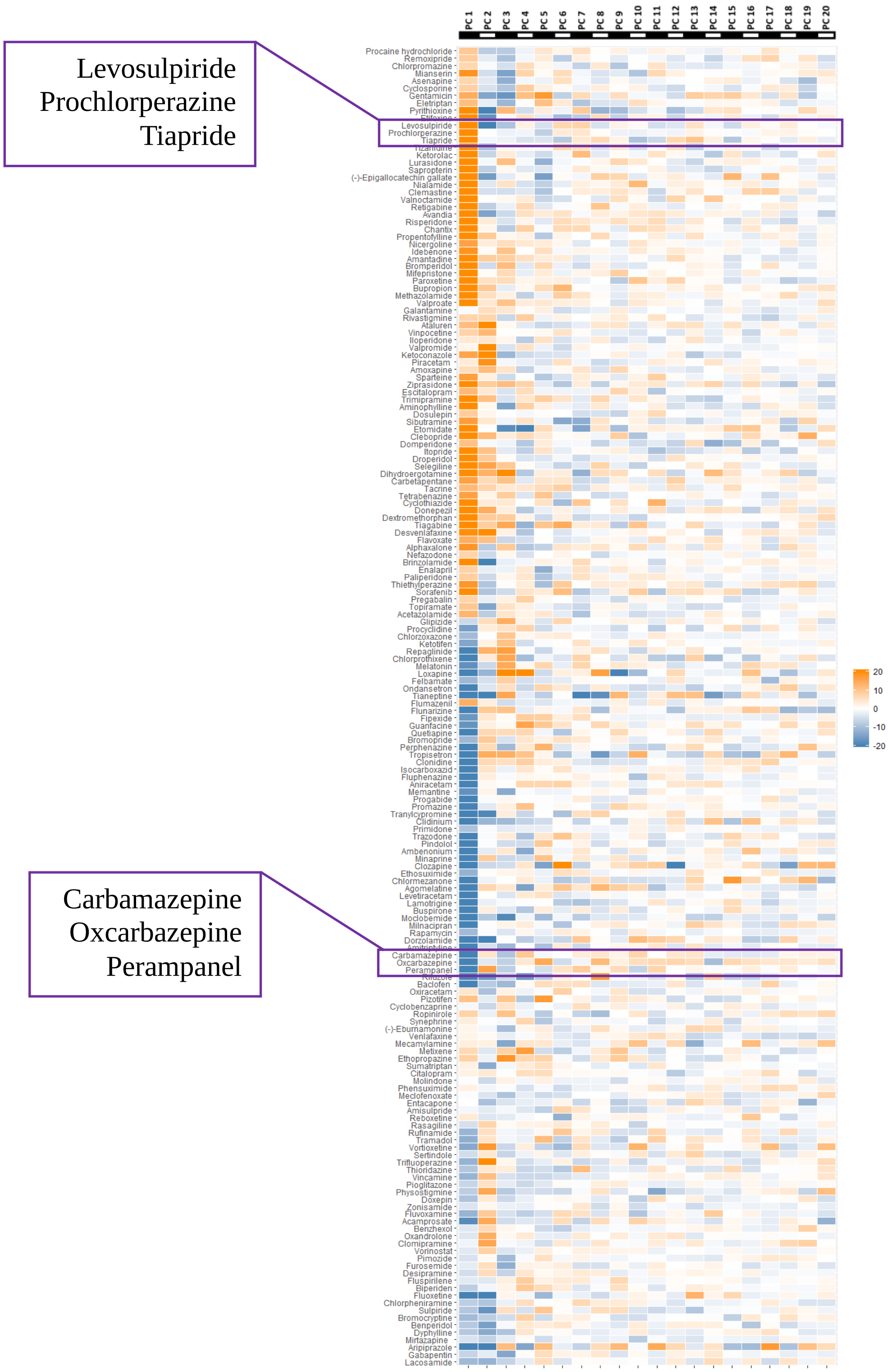

Supplementary Figure 7. The 20-dimensional pheno-prints (featured by the top 20 principal components) for all 179 clinical drugs in the training set.

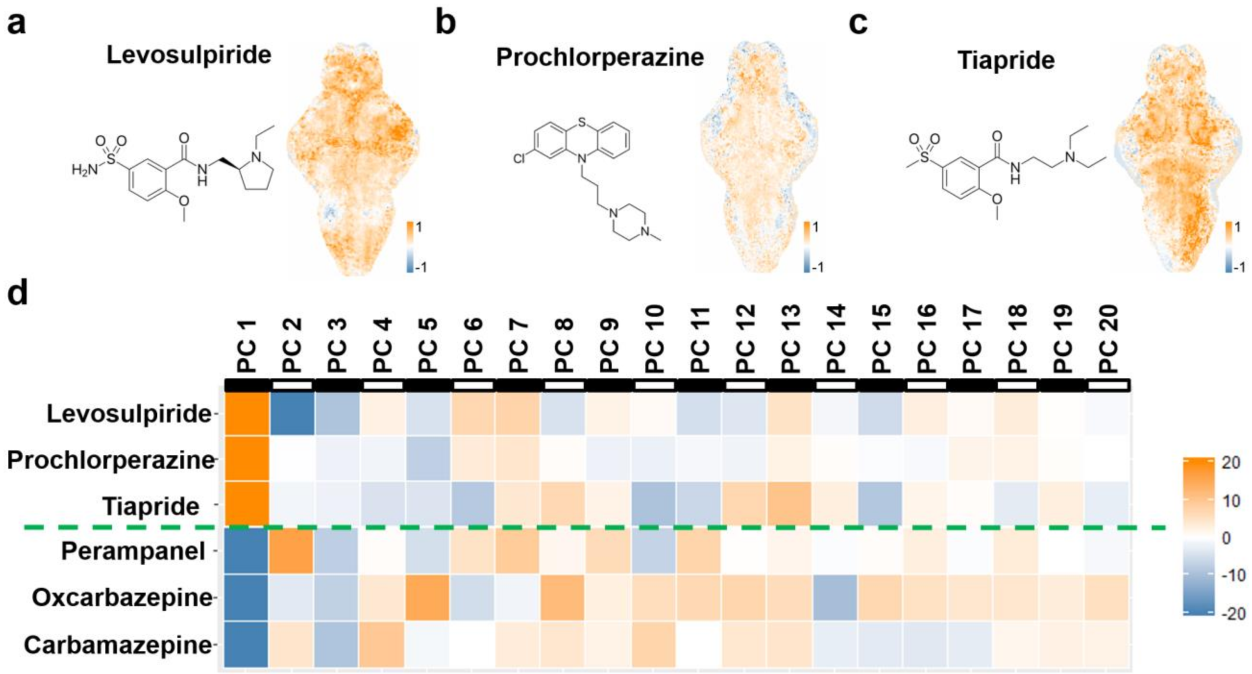

Supplementary Figure 8. Examples of some functionally related drugs sharing similar pheno-prints. (**a-c**) The chemical structures and T-score BAMs of three drugs with similar pharmacological effects, including (**a**) levosulpiride, (**b**) prochlorperazine and (**c**) tiapride. (**d**) Pheno-prints of different drugs, including levosulpiride, prochlorperazine, tiapride, perampanel, oxcarbazepine and carbamazepine. Their relative locations in the training set are indicated by a box in **Supplementary Figure 7**, and the green dash line indicates the separation of two small subgroups**.**

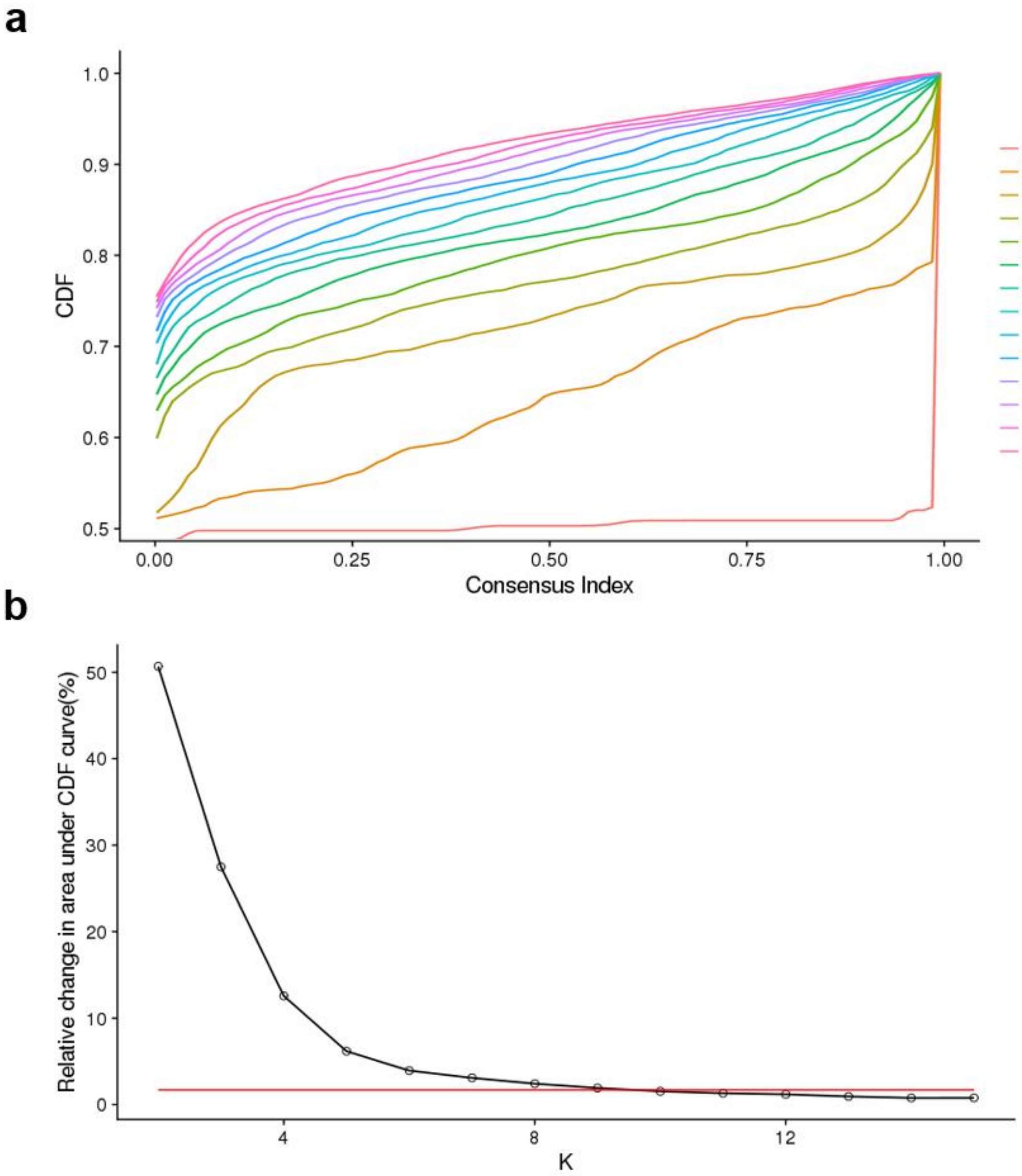

Supplementary Figure 9. Identification of optimal number clusters based on consensus clustering. (**a**) Empirical cumulative distribution function (CDF) for different numbers of clusters ranging from 2 to 15. (**b**) Relative changes in area under the CDF curve as the number of clusters (*K*) increases from 2 to 15. When *K* increases from 10 to 11 and so on, the area under the CDF curve does not increase substantially (<1%), as indicated by the red line.

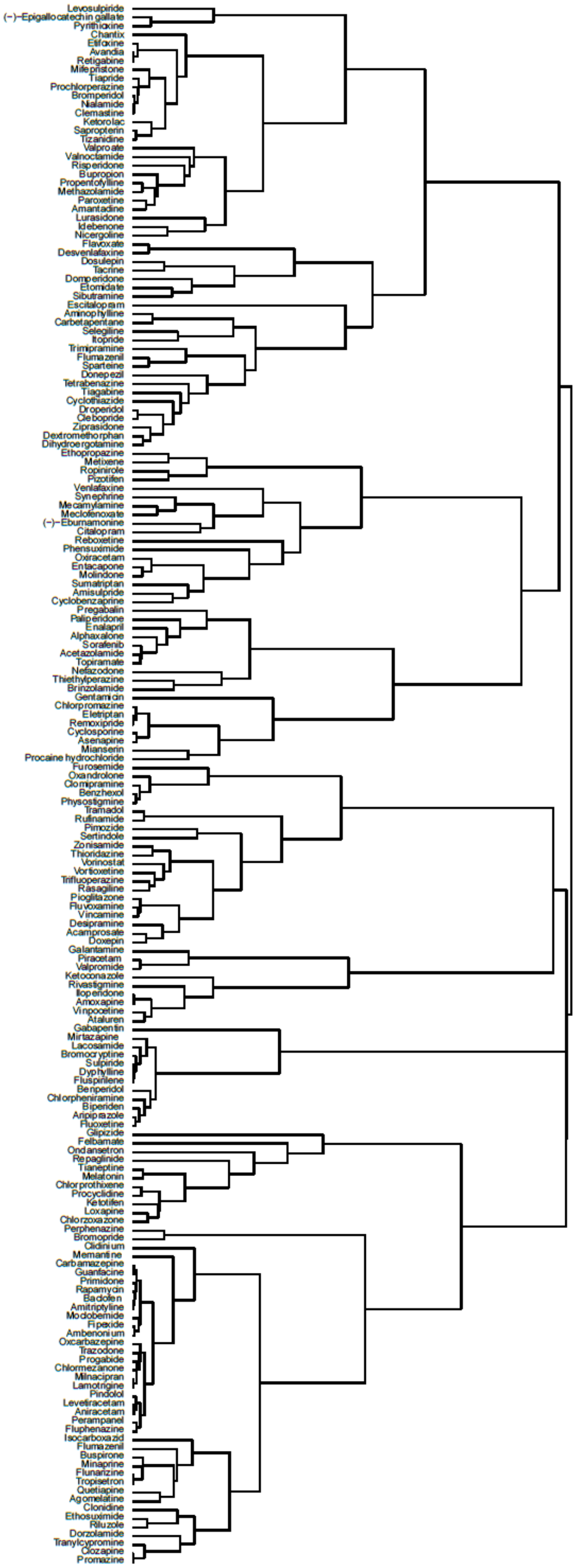

Supplementary Figure 10. The complete dendrogram (with drug names) showing the result of consensus clustering.

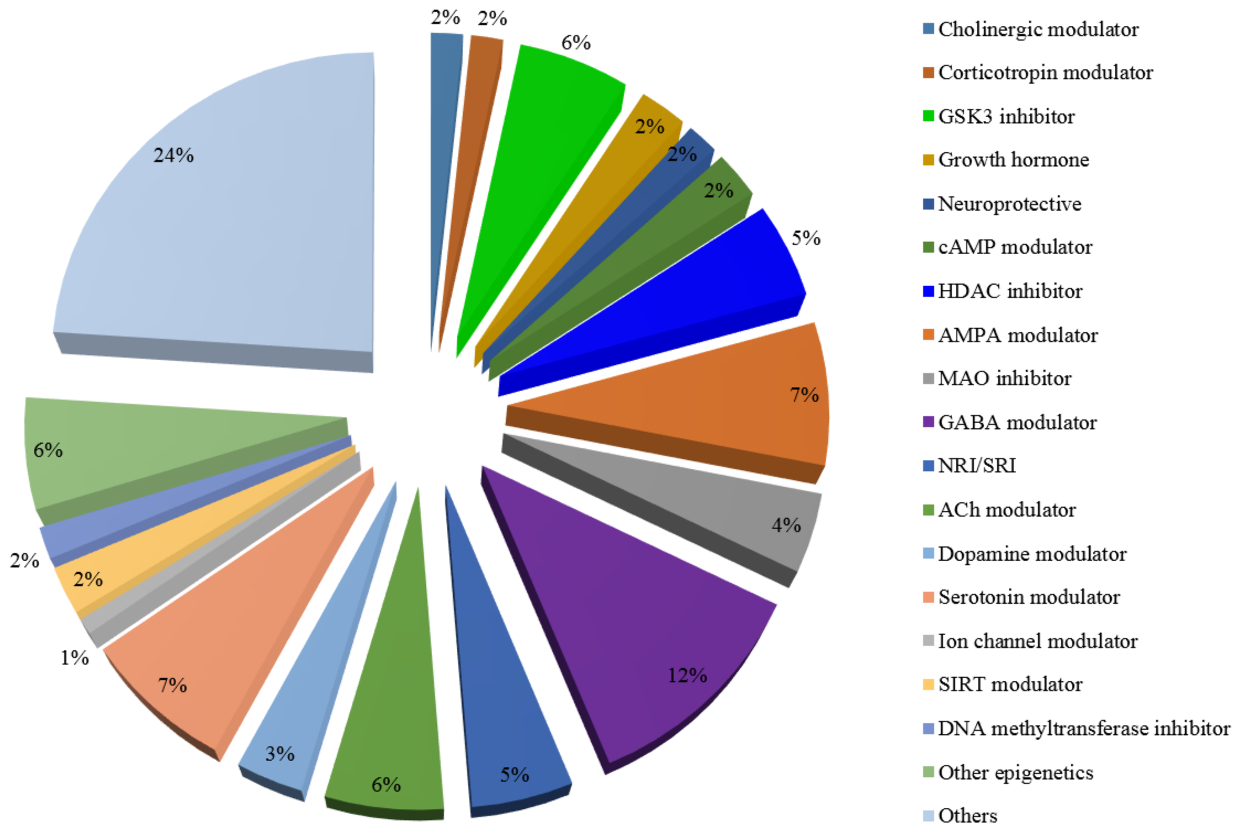

Supplementary Figure 11. The diversity of the test set containing 121 non-clinical compounds as indicated by the pre-annotated molecular targets.

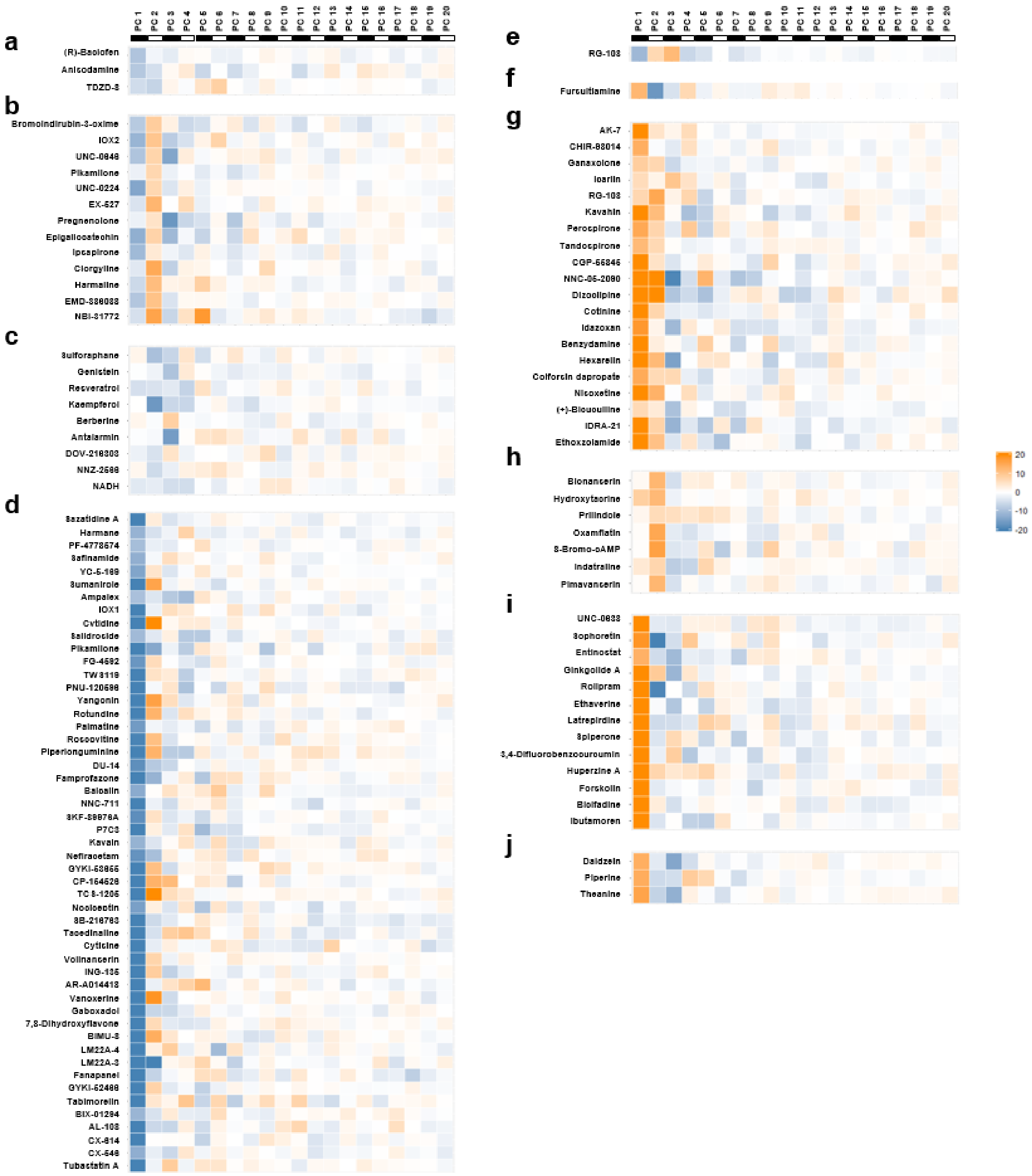

Supplementary Figure 12. The grouped pheno-prints of the 10 predicted clusters of the test set containing 121 non-clinical compounds. (**a-j**) The phenol-prints of predicted cluster 1 to cluster 10.

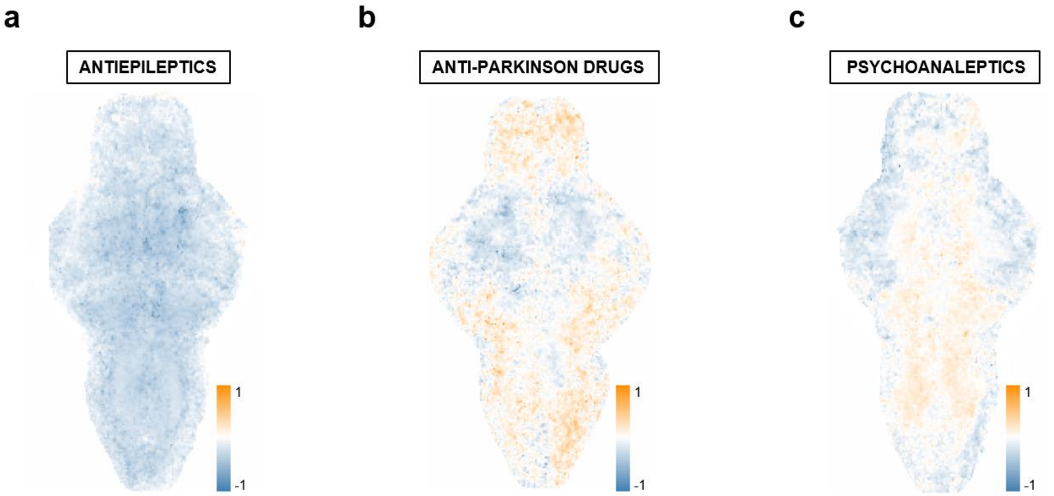

Supplementary Figure 13. The functional signatures generated from the signature sub-group in (**a**) cluster 4, (**b**) cluster 3, and (**c**) cluster 8, which are significantly associated with functional ATC categories as indicated in each panel.

a

Compounds	Classification: First Trial	Classification: Reliability Test
CX-614	Class 4	Class 4
Ethoxzolamide	Class 7	Class 7
IDRA-21	Class 7	Class 7
Bicifadine	Class 9	Class 9
LM22A-3	Class 4	Class 4
LM22A-4	Class 4	Class 4
BIMU-8	Class 4	Class 4
Gaboxadol	Class 4	Class 4
Nisoxetine	Class 7	Class 7
Huperzine A	Class 9	Class 9
Volinanserin	Class 4	Class 4
Cytisine	Class 4	Class 4
SB 216763	Class 4	Class 4
CP-154,526	Class 4	Class 4
Spiperone	Class 9	Class 9
Rolipram	Class 9	Class 9
NNC 711	Class 4	Class 4
Tandospirone	Class 7	Class 7
(+)-Bicuculline	Class 3	Class 7
Ibutamoren	Class 7	Class 9

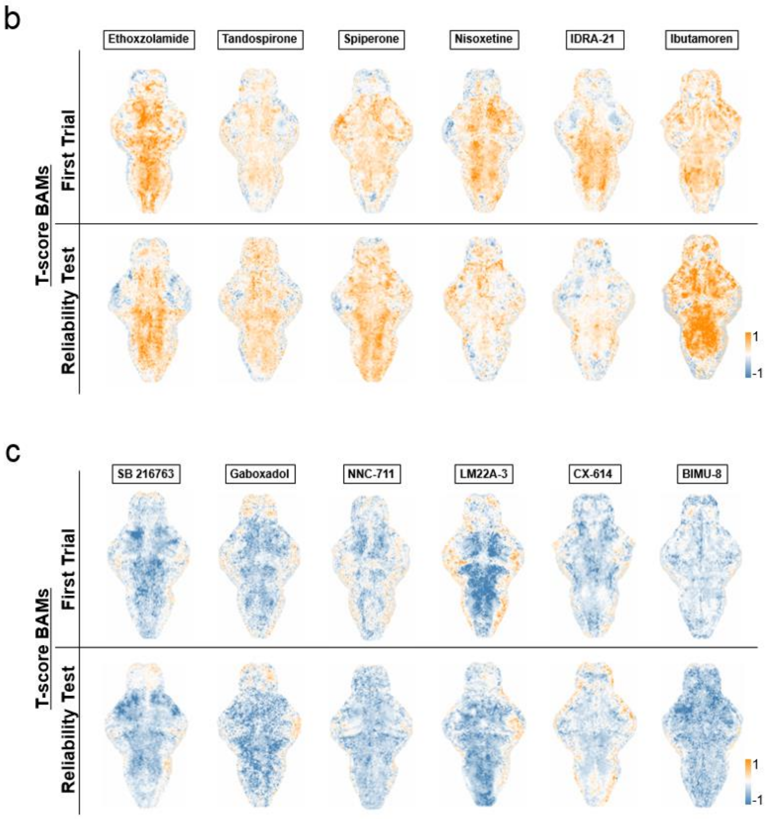

Supplementary Figure 14. The reliability test of repeating and predicting a group of compounds that were already in the test set. (**a**) Comparison of the classification based on the T-score BAMs from the first trial and the reliability test. (**b** - **c**) Examples of T-score BAMs acquired from the first trial and the reliability test for some compounds with BAMs showing (**b**) enhanced neural activity or (**c**) silenced neural activity.

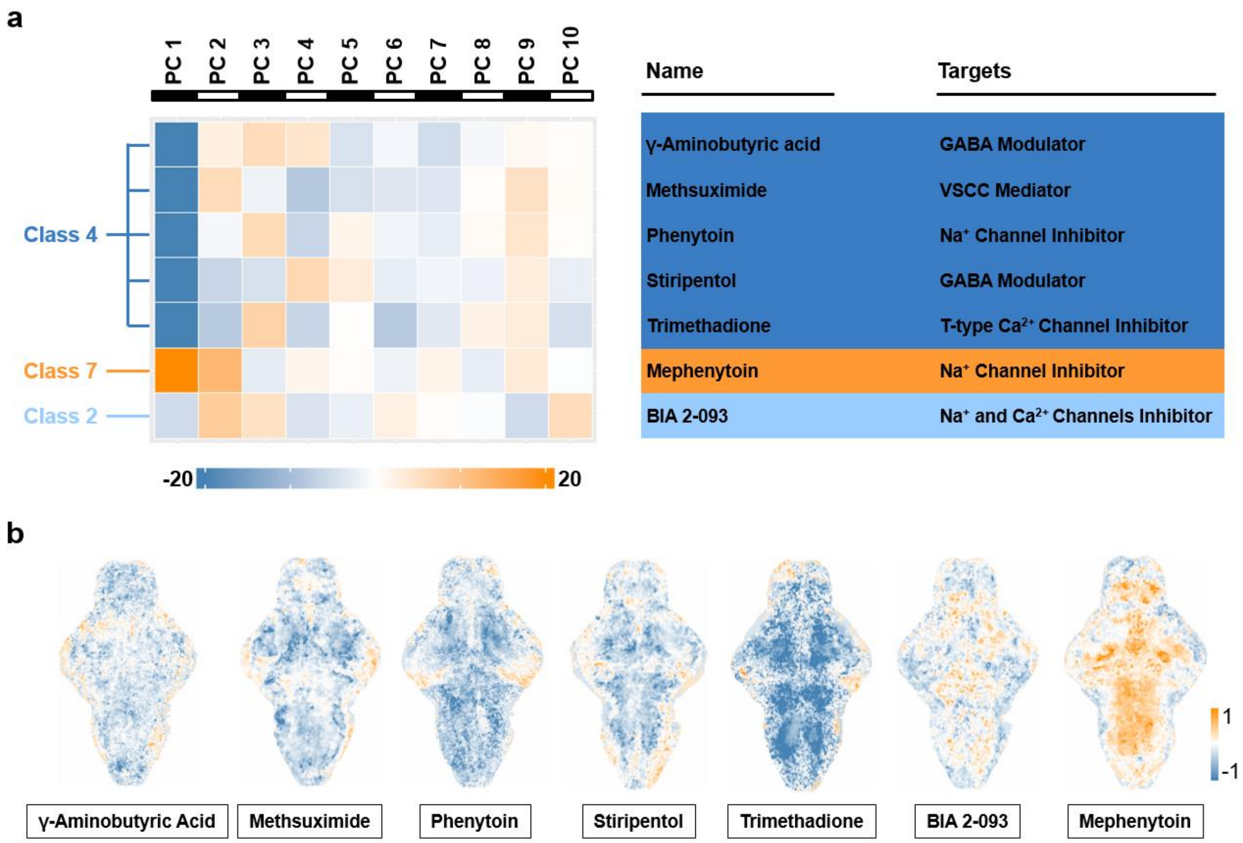

Supplementary Figure 15. Validation of the prediction strategy using extra clinical antiepileptic drugs that were not in the training set. (**a**) Classification of the 7 drugs using their pheno-prints derived from associated T-score BAMs. The dark-blue shaded table shows the drugs with correct predictions. PC, principal components. (**b**) The T-score BAMs of the seven tested antiepileptic drugs.

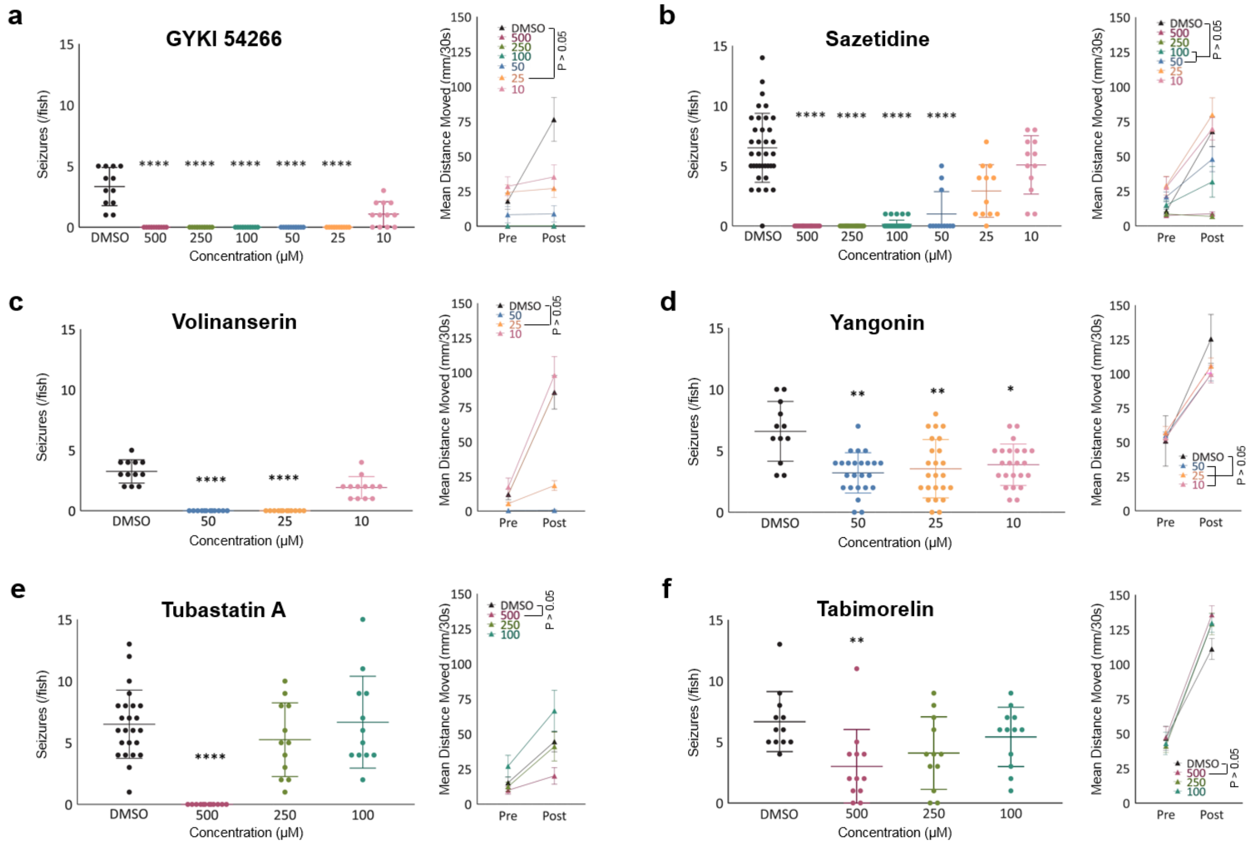

Supplementary Figure 16. More hit compounds identified from the behavioral test using a PTZ seizure animal model. Seizure count over 15 minutes in larvae treated with 5 mM PTZ after 4 hour incubation with (**a**) GYKI 54266, (**b**) Sazetidine, (**c**) Volinanerin, (**d**) Yangonin, (**e**) Tubastatin A, and (**f**) Tabimorelin at different concentrations ranging from 10 to 500 µM, compared to control groups only treated with DMSO. The statistical analysis of the larvae's locomotive behavior before and after a 4 hour pre-treatment with the compounds were also included in each panel, compared to controls (DMSO). Larval behavior and movement was monitored for 15 minutes after a 2 minute light stimulation. Error bars indicate s.e.m., n = 12, ****p < 0.0001, ***p < 0.001, **p < 0.01, *p < 0.05 by one way ANOVA.

SUPPLEMENTARY TABLES

Supplementary Table 1. List of the non-clinical compounds predicted to be Class 4 and were ranked by their correlation to the signature subgroup of cluster 4 (associated with N03:Anti-epileptics ATC category). 14 out of the top 30 compounds (green shaded) were found to have relevant literature supports of having anticonvulsant effects.

Rank	Compound Name	Targets
1	Volinanserin	5-HT2A receptor Inhibitor
2	Sb 216763	GSK3 inhibitor
3	Pikamilone	GABA prodrug
4	Sazetidine A	ACh partial Agonist
5	Gaboxadol	GABAA receptor Agonist
6	AR-A014418	GSK3 Inhibitor
7	IOX1	2OG oxygenase Inhibitor
8	Tubastatin A	HDAC6 Inhibitor
9	TWS119	GSK3 Inhibitor
10	Tabimorelin	Growth hormone secretagogue
11	GYKI-52466	AMPA Modulator
12	Rotundine	D1/D2 Dopaminergic receptors Inhibitor
13	CI-994	HDAC Inhibitor
14	ING-135	GSK3 Inhibitor
15	Yangonin	CB1 receptor Ligand
16	GYKI-53655	AMPA Modulator
17	NNC-711	GABA uptake Inhibitor, Anticonvulsant
18	Fanapanel	AMPA Modulator
19	CX-614	Ampakine, Antidepressant
20	P7C3	Neuroprotective
21	7,8-Dihydroxyflavone	TrkB Agonist
22	AL-108	Neuroprotective
23	PNU-120596	positive allosteric a7 ACh Modulator
24	Sumanirole	D2 receptor Agonist
25	Piperlonguminine	Inhibitor of A-beta production, Natural product
26	YC-5-169	HDAC Inhibitor
27	Vanoxerine	DRI
28	SKF-89976A	GABA Modulator
29	Roscovitine	CDK5 Inhibitor
30	LM22A-4	BDNF mimetics
31	Cytisine	Nicotinic acetylcholine receptor Agonist
32	TCS 1205	GABAA a2 Agonist and GABAA a1 partial Agonist
33	BIMU-8	5-HT4 receptor selective Agonist
34	CP-154,526	CRHR1 Inhibitor
35	Palmatine	AChE Inhibitor, alkaloid
36	DU-14	Steroid sufatase Inhibitor
37	FG-4592	HIF a prolyl hydroxylase Inhibitor
38	Cytidine	Memory Enhancer
39	CX-546	Ampakine
40	LM22A-3	BDNF mimetics
41	Safinamide	MAOI
42	PF 4778574	AMPA Modulator
43	Famprofazone	NSAID
44	Nefiracetam	GABAergic, AChnergic, MNAergic Modulator
45	Harmane	MAOI
46	Nociceptin	Neuropeptide
47	Kavain	Na^{+} and Ca^{2+} channels Inhibitor
48	BIX-01294	HKMT Inhibitor
49	Baicalin	prolyl endopeptidase Inhibitor
50	Ampalex	Ampakine
51	Salidroside	Anxioliytic

Supplementary Table 2. The relative ranking of the 5 extra drugs (blue shaded) when pooled with all predicted Class 4 non-clinical compounds. These 5 drugs were not in the training set and were properly predicted to be Class 4 (potent N03) in the reliability test using 7 extra clinical drugs as positive validation inputs.

Rank	Drug Name	Mechanism of Action & Targets
1	Volinanserin	5-HT2A receptor Inhibitor
2	SB 216763	GSK3 Inhibitor
3	Pikamilone	GABA prodrug
4	Sazetidine A	ACh partial Agonist
5	Gaboxadol	GABAA receptor Agonist
6	AR-A014418	GSK3 Inhibitor
7	IOX1	2OG oxygenase Inhibitor
8	Tubastatin A	HDAC6 Inhibitor
9	TWS119	GSK-3ß Inhibitor
10	Tabimorelin	Growth hormone secretagogue
11	GYKI-52466	AMPA Modulator
12	Trimethadione	T-type Ca2+ Channel Inhibitor
13	Rotundine	D1/D2 Dopaminergic receptors Inhibitor
14	Tacedinaline	HDAC Inhibitor
15	ING-135	GSK3 Inhibitor
16	Stiripentol	GABA Modulator
17	Yangonin	CB1 receptor Ligand
18	GYKI-53655	AMPA Modulator
19	Phenytoin	Na+ Channel Inhibitor
20	NNC 711	GABA uptake Inhibitor, Anticonvulsant
21	Fanapanel	AMPA Modulator
22	CX-614	Ampakine, Antidepressant
23	P7C3	Neuroprotective
24	7,8-Dihydroxyflavone	TrkB agonist
25	AL-108	Neuroprotective
26	PNU-120596	positive allosteric a7 ACh modulator
27	Methsuximide	VSCC Mediator
28	Sumanirole	D2 receptor Agonist
29	Piperlonguminine	Inhibitor of A-beta production, Natural product
30	YC-5-169	HDAC3 Inhibitor
31	Vanoxerine	DRI
32	SKF 89976A	GABA uptake Inhibitor
33	γ-Aminobutyric acid	GABA Modulator
34	Roscovitine	CDK5 Inhibitor
35	LM22A-4	BDNF mimetics
36	Cytisine	Nicotinic acetylcholine receptor Agonist
37	TCS 1205	GABAA a2 agonist and GABAA a1 partial Agonist
38	BIMU-8	5-HT4 receptor selective Agonist
39	CP-154,526	CRHR1 Inhibitor
40	Palmatine	AChE Inhibitor, alkaloid
41	DU-14	Steroid sufatase Inhibitor
42	FG-4592	HIF a prolyl hydroxylase Inhibitor
43	Cytidine	Memory Enhancer
44	CX-546	Ampakine
45	LM22A-3	BDNF mimetics
46	Safinamide	MAOI
47	PF 4778574	AMPA Modulator
48	Famprofazone	NSAID
49	Nefiracetam	GABAergic, AChnergic, MNAergic Modulator
50	Harmane	MAO Inhibitor
51	Nociceptin	Neuropeptide
52	Kavain	Na+ and Ca2+ channels Inhibitor
53	BIX-01294	HKMT Inhibitor
54	Baicalin	prolyl endopeptidase Inhibitor
55	Ampalex	Ampakine
56	Salidroside	Anxiolytic

Supplementary Table 3. List of compounds tested in the behavioral assay in larval zebrafish using a PTZ seizure model. The blue arrows indicate the hit compounds with anti-seizure efficacy.

	Correlation to Cluster 4-N03 Signature	Drug Name	Molecular Targets
→	0.99	Volinanserin	5-HT2A receptor Inhibitor
	0.96	SB 216763	GSK3 Inhibitor
	0.95	Pikamilone	GABA prodrug
→	0.95	Sazetidine A	ACh partial Agonist
	0.95	Gaboxadol	GABAA receptor Agonist
	0.94	AR-A014418	GSK3 Inhibitor
	0.94	IOX1	2OG oxygenase Inhibitor
→	0.94	Tubastatin A	HDAC6 Inhibitor
→	0.94	Tabimorelin	Growth hormone secretagogue
→	0.93	GYKI-52466	AMPA Modulator
→	0.92	Yangonin	CB1 receptor Ligand
→	0.91	NNC 711	GABA uptake Inhibitor, Anticonvulsant
	0.9	P7C3	Neuroprotective
	0.89	7,8-Dihydroxyflavone	TrkB agonist
