# Supplementary material for: High-throughput brain activity mapping and machine learning as a foundation for systems neuropharmacology
Source: Nat Commun. 2018 Dec 3;9:5142. doi: 10.1038/s41467-018-07289-5 (PMC6277389; doi:10.1038/s41467-018-07289-5)
Supplement: Supplementary file 2 — Description of Additional Supplementary Files [file 41467_2018_7289_MOESM2_ESM.pdf]

Description of Additional Supplementary Files

File Name: Supplementary Movie 1
Description: The establishment of a PTZ-seizure model in larval zebrafish, as indicated by a comparison of the whole-brain calcium signals in larvae with or without PTZ treatment.

File Name: Supplementary Movie 2
Description: Behavioral recoding and analysis of larval zebrafish in a 96-well plate for validating the prediction of neuropharmacology in the hit compounds.

File Name: Supplementary Data 1
Description: List of clinically used drugs in the training set.

File Name: Supplementary Data 2
Description: List of the non-clinical compounds in the testing set.
