# Supplementary material for: High-throughput brain activity mapping and machine learning as a foundation for systems neuropharmacology
Source: Nat Commun. 2018 Dec 3;9:5142. doi: 10.1038/s41467-018-07289-5 (PMC6277389; doi:10.1038/s41467-018-07289-5)
Supplement: Supplementary file 5 — Supplementary Data 1 [file 41467_2018_7289_MOESM5_ESM.pdf]

Supplementary Data 1. List of clinically used drugs in the training set.

No.	Drug Name	ATC code	Chemical structure
1	Lacosamide	N03AX18	O=C(N[C@@H](C(=O)NCc1ccccc1)COC)C
2	Riluzole	N07XX02	FC(F)(F)Oc1ccc2nc(sc2c1)N
3	Acetazolamide	S01EC	O=S(=O)(c1nnc(s1)NC(=O)C)N
4	Vorinostat	L01XX38	O=C(Nc1ccccc1)CCCCCCC(=O)NO
5	Valproate	N03AG01	O=C(O)C(CCC)CCC
6	Chantix	N07BA03	C1C2CNCC1C3=CC4=NC=CN=C4C=C23.C(C(C(=O)O)O)(C(=O)O)O
7	Gabapentin	N03AX12	O=C(O)CC1(CN)CCCCC1
8	Amitriptyline	N06AA09	c3cc2c(/C(c1c(cccc1)CC2)=C\CCN(C)C)cc3
9	Flavoxate	G04BD02	O=C(c1c(OC(c2ccccc2)=C(C)C3=O)c3ccc1)OCCN4CCCCC4.Cl
10	Acamprosate	N07BB03	CC(NCCCS(O)(=O)=O)=O.[Ca]
11	Tiagabine	N03AG06	CC1=C(/C(C2=C(C=CS2)C)=C\CCN3CCC[C@@H](C(O)=O)C3)SC=C1.Cl
12	Fluvoxamine	N06AB08	COCCCC/C(C1=CC=C(C(F)(F)F)C=C1)=N\OCCN
13	Citalopram	N06AB04	CN(CCC[C@]1(C2=CC=C(C=C2)F)OCC3=C1C=CC(C#N)=C3)C.Br
14	Eletriptan	N02CC06	CN1CCCC1CC2=CNC3=C2C=C(C=C3)CCS(=O)(C4=CC=CC=C4)=O.Br
15	Methazolamide	S01EC05	CN1N=C(S(N)(=O)=O)S/C1=N\C(C)=O
16	Desipramine	N06AA01	CNCCCN1C2=CC=CC=C2CCC3=CC=CC=C13.Cl
17	Gentamicin	D06AX07	CN[C@@H]([C@@H]1CC[C@H]([C@H](O[C@H]2[C@@H](C[C@@H]([C@H]([C@@H]2O)O[C@@H]3OC[C@@](O)([C@@H]([C@H]3O)NC)C)N)N)O1)N)C.O=S(O)(O)=O
18	Reboxetine	N06AX18	CCOC1=CC=CC=C1OC(C2=CC=CC=C2)C3CNCCO3.CS(=O)(O)=O
19	Dextromethorphan	N07XX59	COC1=CC2=C(C=C1)CC3C4CCCCC24CCN3C.Br.O
20	Zonisamide	N03AX15	NS(=O)(CC1=NOC2=CC=CC=C12)=O
21	Topiramate	N03AX11	CC1(O[C@@H]2CO[C@]3(OC(C)(O[C@H]3[C@@H]2O1)C)COS(N)(=O)=O)C
22	Galantamine	N06DA04	COC1=C2O[C@H]3C[C@H](C=C[C@]34CCN(CC(C=C1)=C24)C)O.Br
23	Aripiprazole	N05AX12	ClC1=CC=CC(N2CCN(CC2)CCCCOC3=CC4=C(C=C3)CCC(N4)=O)=C1Cl
24	Dorzolamide	S01EC03	CCN[C@H]1C[C@@H](S(=O)(C2=C1C=C(S(N)(=O)=O)S2)=O)C.Cl
25	Mecamylamine	C02BB01	CN[C@@]1([C@H]2CC[C@@H](C1(C)C)C2)C.Cl
26	Cyclosporine	L04AD01	O=C(N(C)C(C(CC)NC(C(C(O)C(C)C/C=C/C)N(C)C(C(CCC)N(C)C(C(CCCC)N(C)C(C(CCCC)N(C)C(C(C)NC(C(C)NC(C(CCCC)N(C)C(C(CCC)N1)=O)=O)=O)=O)=O)=O)=O)=O)=O)N(C)C(CCCC)C1=O
27	Memantine	N06DX01	CC12CC3CC(C1)(CC(C3)(C2)N)C.Cl
28	Donepezil	N06DA02	COC1=C(C=C2C([C@H](CC2=C1)CC3CCN(CC4=CC=CC=C4)CC3)=O)OC.Cl
29	Risperidone	N05AX08	CC1=C(C(N2CCCCC2=N1)=O)CCN3CCC(C4=NOC5=C4C=CC(F)=C5)CC3
30	Clomipramine	N06AA04	CN(CCCN1C2=CC=CC=C2CCC3=C1C=C(C=C3)Cl)C.Cl
31	Doxepin	N06AA12	CN(CCC=C1C2=CC=CC=C2COC3=CC=CC=C13)C.Cl
32	Carbamazepine	N03AF01	NC(N1C2=CC=CC=C2C=CC3=CC=CC=C13)=O
33	Fluoxetine	N06AB03	CNCC[C@@H](C1=CC=CC=C1)OC2=CC=C(C(F)(F)F)C=C2.Cl
34	Felbamate	N03AX10	NC(OCC(C1=CC=CC=C1)COC(N)=O)=O
35	Rapamycin	L04AA10	CO[C@@H]1C[C@H](C[C@H]([C@@H]2CC([C@@H](/C=C([C@H]([C@H](C([C@@H](C[C@@H](/C=C\C=C\C=C([C@@H](OC)C[C@@H]3CC[C@H]([C@@](O3)(C(C(N4CCCC[C@H]4C(O2)=O)=O)=O)O)C)/C)C)C)=O)OC)O)\C)C)=O)C)CC[C@H]1O
36	Bupropion	N06AX12	C[C@@H](C(C1=CC(Cl)=CC=C1)=O)NC(C)(C)C.Cl
37	Milnacipran	N06AX17	CCN(C([C@]1(C2=CC=CC=C2)C[C@@H]1CN)=O)CC.Cl
38	Venlafaxine	N06AX16	COC1=CC=C([C@@H](C2(CCCCC2)O)CN(C)C)C=C1.Cl
39	Moclobemide	N06AG02	ClC1=CC=C(C(NCCN2CCOCC2)=O)C=C1
40	Pregabalin	N03AX16	CC(CC(CC(O)=O)CN)C
41	Amoxapine	N06AA17	ClC1=CC2=C(C=C1)OC3=CC=CC=C3N=C2N4CCNCC4
42	Asenapine	N05AH05	OC(/C=C\C(O)=O)=O.ClC1=CC2=C(OC3=C([C@@H]4CN(C[C@H]42)C)C=CC=C3)C=C1
43	Mirtazapine	N06AX11	CN1CCN2[C@@H](C3=CC=CC=C3CC4=C2N=CC=C4)C1
44	Cyclobenzaprine	M03BX08	CN(CCC=C1C2=CC=CC=C2C=CC3=CC=CC=C13)C.Cl
45	Tramadol	N02AX02	COC1=CC=CC(C2(CCCCC2CN(C)C)O)=C1.Cl
46	Ziprasidone	N05AE04	ClC1=C(C=C2CC(NC2=C1)=O)CCN3CCN(C4=NSC5=CC=CC=C45)CC3
47	Tianeptine	N06AX14	OC(CCCCCCNC1C2=C(N(S(=O)(C3=C1C=CC(Cl)=C3)=O)C)C=CC=C2)=O
48	Buspirone	N05BE01	O=C1CC2(CC(N1CCCCN3CCN(C4=NC=CC=N4)CC3)=O)CCCC2
49	Paroxetine	N06AB05	FC1=CC=C(C2CCNCC2COC3=CC4=C(C=C3)OCO4)C=C1.Cl
50	Piracetam	N06BX03	O=C(CN1C(CCC1)=O)N

51	Aniracetam	N06BX11	COC1=CC=C(C(N2CCCC2=O)=O)C=C1
52	Cyclothiazide	C03AA09	NS(=O)(C1=C(C=C2NC(C3CC4CC3C=C4)NS(=O)(C2=C1)=O)Cl)=O
53	Lamotrigine	N03AX09	NC1=NC(N)=C(C2=C(C(Cl)=CC=C2)Cl)N=N1
54	Levetiracetam	N03AX14	CC[C@H](C(N)=O)N1CCCC1=O
55	Perampanel	N03AX22	O=C1C(C2=CC=CC=C2C#N)=CC(C3=CC=CC=N3)=CN1C4=CC=CC=C4
56	Rufinamide	N03AF03	NC(C1=CN(CC2=C(C=CC=C2F)F)N=N1)=O
57	Agomelatine	N06AX22	CC(NCCC1=C2C=C(OC)C=CC2=CC=C1)=O
58	Oxandrolone	A14AA08	C[C@@]1(CC[C@H]2[C@@H]3CC[C@H]4CC(OC[C@@]4([C@H]3CC[C@]12C)C)=O)O
59	Fluphenazine	N05AB02	OCCN1CCN(CC1)CCCN2C3=C(C=CC=C3)SC4=C2C=C(C(F)(F)F)C=C4
60	Isocarboxazid	N06AF01	CC1=CC(C(NNCC2=CC=CC=C2)=O)=NO1
61	Tetrabenazine	N05AK01	O=C1C(CN2C(C1)C3=CC(OC)=C(OC)C=C3CC2)CC(C)C
62	Amisulpride	N05AL05	CCN1CCCC1CNC(C2=CC(S(=O)(CC)=O)=C(C=C2OC)N)=O
63	Baclofen	M03BX01	NCC(C1=CC=C(C=C1)Cl)CC(O)=O
64	Brinzolamide	S01EC04	CCN[C@H]1CN(S(=O)(C2=C1C=C(S(N)(=O)=O)S2)=O)CCCOC
65	Chlormezanone	M03BB02	CN1C(S(=O)(CCC1=O)=O)C2=CC=C(C=C2)Cl
66	Entacapone	N04BX02	CCN(C(/C(C#N)=C/C1=CC([N+]([O-])=O)=C(C(O)=C1)O)=O)CC
67	Ethosuximide	N03AD01	CCC1(CC(NC1=O)=O)C
68	Flumazenil	V03AB25	CCOC(C1=C2CN(C(C3=C(N2C=N1)C=CC(F)=C3)=O)C)=O
69	Tacrine	N06DA01	NC1=C2CCCCC2=NC3=C1C=CC=C3.Cl.O
70	Clozapine	N05AH02	CN1CCN(C2=NC3=C(C=CC(Cl)=C3)NC4=C2C=CC=C4)CC1
71	Loxapine	N05AH01	CN1CCN(C2=NC3=C(C=CC=C3)OC4=C2C=C(C=C4)Cl)CC1.O=C(O)CCC(O)=O
72	Melatonin	N05CH01	COC1=CC2=C(C=C1)NC=C2CCNC(C)=O
73	Mianserin	N06AX03	CN1CCN2C(C3=C(C=CC=C3)CC4=C2C=CC=C4)C1.Cl
74	Minaprine	N06AX07	CC1=C(N=NC(C2=CC=CC=C2)=C1)NCCN3CCOCC3.CC4=C(N=NC(C5=CC=CC=C5)=C4)NCCN6CCOCC6.Cl.Cl
75	Physostigmine	S01EB05	[H][C@]12N(CC[C@]1(C3=C(N2C)C=CC(OC(NC)=O)=C3)C)C
76	Avandia	A10BD03	CN(C1=NC=CC=C1)CCOC2=CC=C(C=C2)CC3SC(NC3=O)=O
77	Sumatriptan	N02CC01	O=C(O)CCC(O)=O.CNS(=O)(CC1=CC2=C(C=C1)NC=C2CCN(C)C)=O
78	Chlorprothixene	N05AF03	CN(CCC=C1C2=C(C=CC=C2)SC3=C1C=C(C=C3)Cl)C.Cl
79	Repaglinide	A10BD14	CCOC1=C(C(O)=O)C=CC(CC(N[C@H](C2=C(N3CCCCC3)C=CC=C2)CC(C)C)=O)=C1
80	Ketotifen	R06AX17	CN1CCC(CC1)=C2C3=C(C(CC4=C2C=CC=C4)=O)SC=C3.CC#C[CH]OOO[O]
81	Etomidate	N01AX07	CCOC(C1=CN=CN1C(C2=CC=CC=C2)C)=O
82	Ketoconazole	D01AC08	CC(N1CCN(C2=CC=C(C=C2)OC[C@@H]3CO[C@](O3)(C4=C(C=C(C=C4)Cl)Cl)CN5C=CN=C5)CC1)=O
83	Ambenonium	N07AA30	CC[N+](CCNC(C(NCC[N+](CC)(CC1=C(C=CC=C1)Cl)CC)=O)=O)(CC2=C(C=CC=C2)Cl)CC.CC[N+](CCNC(C(NCC[N+](CC)(CC3=C(C=CC=C3)Cl)CC)=O)=O)(CC4=C(C=CC=C4)Cl)CC.Cl.Cl
84	Valpromide	N03AG02	CCCC(C(N)=O)CCC
85	Retigabine	N03AX21	CCOC(NC1=CC=C(C=C1N)NCC2=CC=C(C=C2)F)=O
86	Valnoctamide	N05CM13	CCC(C(CC)C)C(N)=O
87	Pindolol	C07AA03	CC(NCC(COC1=CC=CC2=C1C=CN2)O)C
88	Rivastigmine	N06DA03	CCN(C(OC1=CC=CC([C@@H](N(C)C)C)=C1)=O)C.CC#C[CH]OOOOO[O]
89	Mifepristone	G03XB01	[H][C@@]12CC[C@@](C#CC)([C@]1(C[C@@H](C3=C4CCC(C=C4CC[C@@]23[H])=O)C5=CC=C(N(C)C)C=C5)C)O
90	Trazodone	N06AX05	ClC1=CC=CC(N2CCN(CC2)CCCN3N=C4C=CC=CN4C3=O)=C1
91	Pioglitazone	A10BD05	CCC1=CN=C(C=C1)CCOC2=CC=C(C=C2)CC3SC(NC3=O)=O.Cl
92	Clonidine	N02CX02	ClC1=CC=CC(Cl)=C1NC2=NCCN2.Cl
93	Chlorzoxazone	M03BB03	ClC1=CC2=C(C=C1)OC(N2)=O
94	Dyphylline	R03DA01	CN1C2=C(C(N(C1=O)C)=O)N(C=N2)CC(CO)O
95	Pizotifen	N02CX01	CN(CC1)CCC1=C(C2=C(CC3)C=CC=C2)C4=C3SC=C4
96	Primidone	N03AA03	CCC1(C2=CC=CC=C2)C(NCNC1=O)=O
97	Vincamine	C04AX07	O=C([C@@](N1C2=C3C=CC=C2)(O)C[C@@]4(CC)CCCN5CCC3=C1[C@]54[H])OC
98	Ondansetron	A04AA01	CN1C2=C(C(C(CC2)CN3C=CN=C3C)=O)C4=C1C=CC=C4
99	Tropisetron	A04AA03	CN1[C@@H]2CC[C@H]1C[C@@H](OC(C3=CNC4=CC=CC=C43)=O)C2.Cl
100	Bromperidol	N05AD06	FC1=CC=C(C(CCCN2CCC(C3=CC=C(Br)C=C3)(CC2)O)=O)C=C1
101	Sibutramine	A10BG03	CC(CC(C1(C2=CC=C(C=C2)Cl)CCC1)N(C)C)C
102	Benperidol	N05AD07	O=C1NC2=CC=CC=C2N1C3CCN(CCCC(C4=CC=C(F)C=C4)=O)CC3
103	(-)-Eburnamonine	C04AX17	O=C1C[C@@](CCC2)(CC)[C@]3([H])N2CCC4=C3N1C5=C4C=CC=C5
104	Bromocryptine	N04BC01	O=S(C)(O)=O.[H][C@@]12CCCN1C([C@@H](N3C([C@](O[C@@]23O)(C(C)C)NC([C@H]4CN([C@@]5(CC6=C(NC7=C6C(C5=C4)=CC=C7)Br)[H])C)=O)=O)CC(C)C)=O

105	Carbetapentane	R05DB05	O=C(C1(C2=CC=CC=C2)CCCC1)OCCOCCN(CC)CC.OC(CC(O)=O)(C(O)=O)CC(O)=O
106	Clemastine	D04AA14	CN1CCC[C@@H]1CCO[C@](C2=CC=CC=C2)(C3=CC=C(C=C3)Cl)C.O=C(/C=C/C(O)=O)O
107	Clidinium	A03CA02	C[N+]12CCC(C(OC(C(C3=CC=CC=C3)(C4=CC=CC=C4)O)=O)C2)CC1.[Br]
108	Dihydroergotamine	N02CA01	[H][C@@]12CCCN1C([C@@H](N3C([C@](NC([C@H]4CN([C@@]5(CC6=CNC7=C6C([C@]5(C4)[H])=CC=C7)[H])C)=O)(O[C@@]23O)C)=O)CC8=CC=CC=C8)=O.OC(C(O)C(O)=O)C(O)=O
109	Dosulepin	N06AA16	CN(C)CC/C=C1C2=CC=CC=C2SCC3=CC=CC=C/13.Cl
110	Selegiline	N04BD01	C[C@@H](N(CC#C)C)CC1=CC=CC=C1.Cl
111	Ethopropazine	N04AA05	CCN(C(CN1C2=C(C=CC=C2)SC3=C1C=CC=C3)C)CC.Cl
112	Meclofenoxate	N06BX01	ClC1=CC=C(OCC(OCCN(C)C)=O)C=C1.Cl
113	Metixene	N04AA03	CN1CCCC(C1)CC2C3=C(C=CC=C3)SC4=C2C=CC=C4.Cl
114	Phensuximide	N03AD02	CN1C(CC(C2=CC=CC=C2)C1=O)=O
115	Procyclidine	N04AA04	OC(C1CCCCC1)(C2=CC=CC=C2)CCN3CCCC3.Cl
116	Chlorpromazine	N05AA01	CN(CCCN1C2=C(C=CC=C2)SC3=C1C=C(C=C3)Cl)C
117	Biperiden	N04AA02	OC(C1CC2CC1C=C2)(C3=CC=CC=C3)CCN4CCCCC4
118	Thiethylperazine	R06AD03	CCSC1=CC2=C(C=C1)SC3=C(N2CCCN4CCN(CC4)C)C=CC=C3.OC(CC(O)=O)C(O)=O
119	Tranylcypromine	N06AF04	N[C@@H]1CC1C2=CC=CC=C2.Cl
120	Glipizide	A10BB07	CC1=CN=C(C(NCCC2=CC=C(S(=O)(NC(NC3CCCCC3)=O)=O)C=C2)=O)C=N1
121	Perphenazine	N05AB03	OCCN1CCN(CC1)CCCN2C3=C(C=CC=C3)SC4=C2C=C(C=C4)Cl
122	Aminophylline	R03DA05	NCCN.CN1C2=C(C(N(C1=O)C)=O)NC=N2.NCCN.CN3C4=C(C(N(C3=O)C)=O)NC=N4
123	Sulpiride	N05AL01	CCN1CCCC1CNC(C2=CC(S(N)(=O)=O)=CC=C2OC)=O
124	Benzhexol	N04AA01	OC(C1CCCCC1)(C2=CC=CC=C2)CCN3CCCCC3.Cl
125	Bromopride	A03FA04	BrC1=C(N)C=C(OC)C(C(NCCN(CC)CC)=O)=C1
126	Amantadine	N04BB01	NC12CC3CC(C2)CC(C1)C3
127	Nialamide	N06AF02	O=C(NCC1=CC=CC=C1)CCNNC(C2=CC=NC=C2)=O
128	Fluspirilene	N05AG01	FC1=CC=C(C(C2=CC=C(C=C2)F)CCCN3CCC4(N(C5=CC=CC=C5)CNC4=O)CC3)C=C1
129	Furosemide	C03CA01	NS(=O)(C1=CC(C(O)=O)=C(C=C1Cl)NCC2=CC=CO2)=O
130	Droperidol	N05AD08	FC1=CC=C(C(CCCN2CCC(N3C(NC4=C3C=CC=C4)=O)=CC2)=O)C=C1
131	Promazine	N05AA03	CN(CCCN1C2=C(C=CC=C2)SC3=C1C=CC=C3)C.Cl
132	Pimozide	N05AG02	FC1=CC=C(C(C2=CC=C(C=C2)F)CCCN3CCC(N4C(NC5=C4C=CC=C5)=O)CC3)C=C1
133	Tiapride	N05AL03	O=C(NCCN(CC)CC)C1=CC(S(=O)(C)=O)=CC=C1OC.Cl
134	Prochlorperazine	N05AB04	O=C(/C=C\C(O)=O)O.CN1CCN(CC1)CCCN2C3=C(SC4=CC=C(C=C24)Cl)C=CC=C3
135	Trimipramine	N06AA06	CC(CN1C2=C(C=CC=C2)CCC3=C1C=CC=C3)CN(C)C.O=C(O)/C=C\C(O)=O
136	Paliperidone	N05AX13	O=C1N2C([C@@H](CCC2)O)=NC(C)=C1CCN3CCC(CC3)C4=NOC5=C4C=CC(F)=C5
137	Quetiapine	N05AH04	OCCOCCN1CCN(C2=NC3=CC=CC=C3SC4=CC=CC=C24)CC1.OC(/C=C/C(O)=O)=O
138	Enalapril	C09AA02	CCOC([C@H](N[C@H](C(N1CCC[C@H]1C(O)=O)=O)C)CCC2=CC=CC=C2)=O
139	Synephrine	C01CA08	CNCC(C1=CC=C(O)C=C1)O
140	Itopride	A03FA07	COC1=C(OC)C=CC(C(NCC2=CC=C(C=C2)OCCN(C)C)=O)=C1.Cl
141	Oxcarbazepine	N03AF02	NC(N1C2=CC=CC=C2CC(C3=CC=CC=C13)=O)=O
142	Iloperidone	N05AX14	COC1=CC(C(C)=O)=CC=C1OCCCN2CCC(C3=NOC4=C3C=CC(F)=C4)CC2
143	Sparteine	C01BA04	N12CCCC[C@H]1[C@H](CN3CCCC[C@@H]43)C[C@H]4C2
144	Sorafenib	L01XE05	CNC(C1=NC=CC(OC2=CC=C(C=C2)NC(NC3=CC(C(F)(F)F)=C(C=C3)Cl)=O)=C1)=O
145	Domperidone	A03FA03	ClC1=CC2=C(N(C(N2)=O)C3CCN(CC3)CCCN4C(NC5=CC=CC=C45)=O)C=C1
146	Clebopride	A03FA06	ClC1=C(N)C=C(OC)C(C(NC2CCN(CC2)CC3=CC=CC=C3)=O)=C1.OC(CC(O)=O)C(O)=O
147	Procaine hydrochloride	C05AD05	CCN(CCOC(C1=CC=C(C=C1)N)=O)CC.Cl
148	(-)-Epigallocatechin gallate	D06BB12	O=C(C1=CC(O)=C(O)C(O)=C1)O[C@@H]2CC3=C(C=C(C=C3O)O)O[C@@H]2C4=CC(O)=C(O)C(O)=C4
149	Ropinirole	N04BC04	CCCN(CCC1=C2CC(NC2=CC=C1)=O)CCC.Cl
150	Idebenone	N06BX13	O=C1C(CCCCCCCCCCO)=C(C)C(C(OC)=C1OC)=O
151	Thioridazine	N05AC02	CSC1=CC2=C(C=C1)SC3=CC=CC=C3N2CCC4CCCCN4C.Cl
152	Nefazodone	N06AX06	CCC1=NN(C(N1CCOC2=CC=CC=C2)=O)CCCN3CCN(C4=CC(Cl)=CC=C4)CC3.Cl

153	Guanfacine	C02AC02	NC(NC(CC1=C(C=CC=C1Cl)Cl)=O)=N.Cl
154	Rasagiline	N04BD02	C#CCN[C@@H]1CCC2=CC=CC=C12
155	Vinpocetine	N06BX18	O=C(C1=C[C@@](CCC2)(CC)[C@@H]3N2CCC4=C3N1C5=CC=CC=C45)OCC
156	Nicergoline	C04AE02	CO[C@]12C[C@H](CN([C@@H]1CC3=CN(C4=CC=CC2=C34)C)C)COC(C5=CC(Br)=CN=C5)=O
157	Fipexide	N06BX05	ClC1=CC=C(OCC(N2CCN(CC3=CC4=C(OCO4)C=C3)CC2)=O)C=C1.Cl
158	Propentofylline	N06BC02	O=C(N1CCCCC(C)=O)N(C)C2=C(N(CCC)C=N2)C1=O
159	Oxiracetam	N06BX07	O=C(CN1C(CC(C1)O)=O)N
160	Pyrithioxine	N06BX02	OC1=C(C)N=CC(CSSCC2=C(CO)C(O)=C(C)N=C2)=C1CO
161	Progabide	N03AG05	NC(CCCN/C(C1=CC=C(C=C1)Cl)=C2C=C(C=CC\2=O)F)=O
162	Ataluren	M09AX03	O=C(C1=CC=CC(C2=NOC(C3=CC=CC=C3F)=N2)=C1)O
163	Sapropterin	A16AX07	CC(O)[C@H](O)C1CNC2=C(N1)C(N=C(N)N2)=O.Cl
164	Trifluoperazine	N05AB06	CN1CCN(CCCN2C3=CC=CC=C3SC4=C2C=C(C=C4)C(F)(F)F)CC1.Cl
165	Flunarizine	N07CA03	FC1=CC=C(C=C1)C(N2CCN(C/C=C/C3=CC=CC=C3)CC2)C4=CC=C(F)C=C4.Cl
166	Levosulpiride	N05AL07	CCN1CCC[C@H]1CNC(C2=C(C=CC(S(N)(=O)=O)=C2)OC)=O
167	Desvenlafaxine	N06AX23	CN(CC(C1(CCCCC1)O)C2=CC=C(C=C2)O)C
168	Chlorpheniramine	R06AB04	CN(CCC(C1=CC=CC=N1)C2=CC=C(C=C2)Cl)C.O=C(O)/C=C\C(O)=O
169	Lurasidone	N05AE05	O=C1[C@H]2[C@@H]3CC[C@H]([C@H]2C(N1C[C@@H]4CCCC[C@H]4CN5CCN(C6=NSC7=CC=CC=C67)CC5)=O)C3.Cl
170	Remoxipride	N05AL04	CCN1CCC[C@H]1CNC(C2=C(C=CC(Br)=C2OC)OC)=O.Cl
171	Ketorolac	M01AB15	OC(C1CCN2C1=CC=C2C(C3=CC=CC=C3)=O)=O
172	Tizanidine	M03BX02	ClC1=C(C2=NSN=C2C=C1)NC3=NCCN3.Cl
173	Sertindole	N05AE03	FC1=CC=C(N2C=C(C3=C2C=CC(Cl)=C3)C4CCN(CC4)CCN5CCNC5=O)C=C1
174	Molindone	N05AE02	CCC1=C(NC2=C1C(C(CC2)CN3CCOCC3)=O)C.Cl
175	Vortioxetine	N06AX26	CC1=C(SC2=C(C=CC=C2)N3CCNCC3)C=CC(C)=C1.Br
176	Escitalopram	N06AB10	CN(CCC[C@@]1(C2=CC=C(C=C2)F)OCC3=C1C=CC(C#N)=C3)C.O=C(O)C(O)=O.O
177	Flumazenil	V03AB25	CCOC(C1=C2CN(C(C3=C(N2C=N1)C=CC(F)=C3)=O)C)=O
178	Alphaxalone	N01AX05	O=C1[C@H]2[C@@H](CC[C@H]3C[C@@H](CC[C@]23C)O)[C@@H]4CC[C@@H]([C@]4(C1)C)C(C)=O
179	Etifoxine	N05BX03	ClC1=CC=C2N=C(OC(C)(C2=C1)C3=CC=CC=C3)NCC.Cl
