# Supplementary material for: High-throughput brain activity mapping and machine learning as a foundation for systems neuropharmacology
Source: Nat Commun. 2018 Dec 3;9:5142. doi: 10.1038/s41467-018-07289-5 (PMC6277389; doi:10.1038/s41467-018-07289-5)
Supplement: Supplementary file 6 — Supplementary Data 2 [file 41467_2018_7289_MOESM6_ESM.pdf]

Supplementary Data 2. List of the non-clinical compounds in the testing set.

No.	Drug Name	ATC code	Chemical Structure
1	Tubastatin A	no entry	O=C(C1=CC=C(CN2C3=C(C4=C2C=CC=C4)CN(C)CC3)C=C1)NO.Cl
2	NADH	no entry	NC(C1=CN([C@H]2O[C@@H](C(C2O)O)COP(OP(OC[C@@H]3O[C@H](N4C=NC5=C(N=CN=C45)N)C(C3O)O)(O[K])=O)(O[K])=O)C=CC1)=O
3	CX-546	no entry	O=C(C1=CC2=C(OCCO2)C=C1)N3CCCCC3
4	CX-614	no entry	O=C(C1=CC2=C(OCCO2)C=C1O3)N4C3CCC4
5	Ibutamoren	no entry	O=S(C)(N1CC2(CCN(C(C(COCC3=CC=CC=C3)NC(C(C)(C)N)=O)=O)CC2)C4=C1C=CC=C4)=O
6	AL-108	no entry	Asn-Ala-Pro-Val-Ser-Ile-Pro-Gln
7	Ethoxzolamide	no entry	CCOC1=CC2=C(N=C(S(N)(=O)=O)S2)C=C1
8	NBI-31772	no entry	O=C(C1=CC2=C(C(C(C3=CC=C(O)C(O)=C3)=O)=N1)C=C(O)C(O)=C2)O.O.O
9	NNZ-2566	no entry	O=C(CN)N1[C@@](C(N[C@H](C(O)=O)CCC(O)=O)=O)(C)CCC1.Cl
10	Theanine	no entry	O=C([C@H](CCC(NCC)=O)N)O
11	IDRA-21	no entry	CC(N1)NC2=CC=C(C=C2S1(=O)=O)Cl
12	BIX-01294	no entry	COC1=CC2=NC(N3CCN(CCC3)C)=NC(NC4CCN(CC4)CC5=CC=CC=C5)=C2C=C1OC.Cl.Cl.Cl
13	Tabimorelin	no entry	CC(N)(C/C=C/C(N([C@H](CC1=CC=C2C=CC=CC2=C1)C(N([C@H](CC3=CC=CC=C3)C(NC)=O)C)=O)C)=O)C.[C]#[C].O.O
14	Pimavanserin	no entry	CC(COC1=CC=C(CNC(N(C2CCN(CC2)C)CC3=CC=C(C=C3)F)=O)C=C1)C
15	DOV-216,303	no entry	ClC1=CC=C(C23CNCC2C3)C=C1Cl.Cl
16	Bicifadine	no entry	CC1=CC=C(C23CNCC2C3)C=C1.Cl
17	Indatraline	no entry	CNC1CC(C2=CC=C(Cl)C(Cl)=C2)C3=C1C=CC=C3.Cl
18	GYKI-52466	no entry	CC1=NN=C(C2=CC=C(N)C=C2)C3=CC4=C(OCO4)C=C3C1.Cl
19	Fanapanel	no entry	FC(F)(F)C1=CC(NC(C(N2CP(O)(O)=O)=O)=O)=C2C=C1N3CCOCC3
20	BIMU-8	no entry	O=C(N1C2=CC=CC=C2N(C(C)C)C1=O)NC3C[C@@](N4C)([H])CC[C@@]4([H])C3.Cl
21	7,8-Dihydroxyflavone	no entry	O=C1C=C(C2=CC=CC=C2)OC3=C1C=CC(O)=C3O
22	Gaboxadol	no entry	OC1=NOC2=C1CCNC2.Cl
23	LM22A-3	no entry	CC(NC1=CC=C(/C=C(C2=NN(CCO)C(N)=C2C#N)/C#N)C=C1)=O
24	LM22A-4	no entry	O=C(C1=CC(C(NCCO)=O)=CC(C(NCCO)=O)=C1)NCCO
25	(+)-Bicuculline	no entry	CN1CCC2=CC3=C(C=C2[C@H]1[C@@H]4OC(C5=C4C=CC6=C5OCO6)=O)OCO3
26	Nisoxetine	no entry	CNCCC(OC1=CC=CC=C1OC)C2=CC=CC=C2.Cl
27	Vanoxerine	no entry	FC1=CC=C(C=C1)C(OCCN2CCN(CC2)CCCC3=CC=CC=C3)C4=CC=C(C=C4)F.Cl
28	Forskolin	no entry	[H][C@@]1(CCC(C)([C@@]2([C@](O)([C@](OC(C)=O)([C@]3(O[C@](CC([C@@]3([C@@]12C)O)=O)(C=C)C)C)[H])[H])[H])C)O
29	AR-A014418	no entry	O=C(NCC1=CC=C(OC)C=C1)NC2=NC=C([N+]([O-])=O)S2
30	ING-135	no entry	O=C1C(C2=CN(C)C3=CC=C(Br)C=C32)=C(C4=COC5=C4C=CC=C5)C(N1)=O
31	Huperzine A	no entry	CC1=C[C@H]2CC(N3)=C([C@@](C1)(/C2=C\C)N)C=CC3=O
32	Volinanserin	no entry	COC1=CC=CC([C@@H](C2CCN(CC2)CCC3=CC=C(C=C3)F)O)=C1OC
33	8-Bromo-cAMP	no entry	BrC1=NC2=C(N)N=CN=C2N1C3OC4COP(OC4C3O)(O)=O.[Na]
34	Bromoindirubin-3-oxime	no entry	BrC(C=C1)=CC(NC/2=O)=C1C2=C3C(NO)=C4C=CC=CC4=N/3
35	Cytisine	no entry	O=C1C=CC=C2N1C[C@@H]3CNC[C@H]2C3
36	3,4-Difluorobenzocurcumin	no entry	O=C(/C(C(/C=C/C1=CC=C(O)C(COC)=C1)=O)=C\C2=CC=C(F)C(F)=C2)/C=C/C3=CC(OC)=C(O)C=C3
37	Tacedinaline	no entry	O=C(NC1=CC=CC=C1N)C2=CC=C(NC(C)=O)C=C2
38	TDZD-8	no entry	O=C(N1CC2=CC=CC=C2)N(C)SC1=O
39	Oxamflatin	no entry	O=C(NO)/C=C/C#CC1=CC=CC(NS(=O)(C2=CC=CC=C2)=O)=C1
40	EMD-386,088	no entry	ClC1=CC2=C(NC(C)=C2C3=CCNCC3)C=C1.Cl
41	SB 216763	no entry	O=C(C(C1=CC=C(Cl)C=C1Cl)=C2C3=CN(C)C4=C3C=CC=C4)NC2=O
42	Colforsin dapropate	no entry	C=C[C@]1(CC(C2([C@@](O1)([C@H]([C@H]([C@@]3([H])C(C)(CC[C@@H]([C@@]23C)O)C)OC(CCN(C)C)=O)OC(C)=O)C)O)=O)C.Cl
43	RG-108	no entry	O=C1C2=C(C=CC=C2)C(N1[C@H](C(O)=O)CC3=CNC4=CC=CC=C43)=O
44	Antalarmin	no entry	CC1=C(N2C(C)=C(C)C3=C2N=C(C)N=C3N(CCCC)CC)C(C)=CC(C)=C1.Cl
45	TCS 1205	no entry	O=C(N[C@H](C)C1=CC=CC=C1)C(C2=CNC3=C2C=C([N+]([O-])=O)C=C3)=O
46	CP-154,526	no entry	CCCCN(C1=NC(C)=NC2=C1C(C)=CN2C3=C(C)C=C(C)C=C3C)CC.Cl
47	GYKI-53655	no entry	CC1N(C(NC)=O)N=C(C2=CC=C(N)C=C2)C3=CC4=C(OCO4)C=C3C1.Cl

48	Nefiracetam	no entry	O=C1N(CCC1)CC(NC2=C(C)C=CC=C2C)=O
49	Hexarelin	no entry	NCCCC[C@@H](C(N)=O)NC([C@@H](CC1=CC=CC=C1)NC([C@H](CC2=CNC3=CC=CC=C23)NC([C@H](C)NC([C@@H](CC4=C(C)NC5=CC=CC=C45)NC([C@H](CC6=CNC=N6)N)=O)=O)=O)=O)=O
50	Nociceptin	no entry	O=C(NCC(NCC(N[C@@H](CC1=CC=CC=C1)C(N[C@@H]([C@H](O)C)C(NCC(N[C@@H](C)C(N[C@@H](CCCNC(N)=N)C(N[C@@H](CCCCN)C(N[C@@H](CO)C(N[C@@H](C)C(N[C@@H](CCCNC(N)=N)C(N[C@@H](CCCCN)C(N[C@@H](CC(C)C)C(N[C@@H](C)C(N[C@@H](CC(N)=O)C(N[C@@H](CCC(N)=O)C(O)=O)=O)=O)=O)=O)=O)=O)=O)=O)=O)=O)=O)=O)=O)=O)=O)[C@H](CC2=CC=CC=C2)N
51	Fursultiamine	no entry	O=CN(CC1=C(N)N=C(C)N=C1)C(C)=C(CCO)SSCC2OCCC2
52	Spiperone	no entry	FC1=CC=C(C(CCCN2CCC3(C(NCN3C4=CC=CC=C4)=O)CC2)=O)C=C1
53	Harmaline	no entry	CC1=NCCC2=C1NC3=C2C=CC(OC)=C3
54	Kavain	no entry	O=C1C=C(OC)C[C@H](/C=C/C2=CC=CC=C2)O1
55	P7C3	no entry	OC(CN1C2=C(C3=C1C=CC(Br)=C3)C=C(Br)C=C2)CNC4=CC=CC=C4
56	Latrepirdine	no entry	CC1=CC=C(CCN2C3=C(CN(C)CC3)C4=C2C=CC(C)=C4)C=N1.CC5=CC=C(CCN6C7=C(CN(C)CC7)C8=C6C=CC(C)=C8)C=N5.Cl.Cl
57	Benzydamine	no entry	CN(C)CCCOC1=NN(CC2=CC=CC=C2)C3=C1C=CC=C3.Cl
58	Berberine	no entry	COC(C=CC1=C2C=[N+](CC3)C(C4=C3C=C5C(OCO5)=C4)=C1)=C2OC.[Cl]
59	Clorgyline	no entry	ClC1=CC(Cl)=CC=C1OCCCN(C)CC#C.Cl
60	Ethaverine	no entry	CCOC1=CC2=C(C=C1OCC)C=CN=C2CC3=CC=C(OCC)C(OCC)=C3.Cl
61	Ginkgolide A	no entry	C[C@@H]1C(O[C@H]2C[C@@]34[C@H]5C[C@H]([C@@]36[C@H](C(O[C@H]6O[C@]4([C@@]12O)C(O5)=O)=O)O)C(C)(C)C)=O
62	Idazoxan	no entry	C1(C2COC3=CC=CC=C3O2)=NCCN1.Cl
63	Pirlindole	no entry	CC1=CC2=C(N3CCNC4C3=C2CCC4)C=C1.CS(O)(=O)=O
64	Rolipram	no entry	O=C1NCC(C2=CC=C(C(OC3CCCC3)=C2)OC)C1
65	Dizocilpine	no entry	C[C@@]12C3=CC=CC=C3[C@H](N2)CC4=CC=CC=C14.O=C([O-])/C=C\C([O-])=O
66	SKF 89976A	no entry	O=C(C1CN(CCC=C(C2=CC=CC=C2)C3=CC=CC=C3)CCC1)O.Cl
67	NNC 05-2090	no entry	OC1(C2=CC=CC=C2OC)CCN(CCCN3C4=C(C5=C3C=CC=C5)C=CC=C4)CC1.Cl
68	NNC 711	no entry	O=C(C1=CCCN(CCON=C(C2=CC=CC=C2)C3=CC=CC=C3)C1)O.Cl
69	Cotinine	no entry	O=C1N([C@@H](CC1)C2=CC=CN=C2)C
70	Famprofazone	no entry	O=C1C(C(C)C)=C(CN(C)C(CC2=CC=CC=C2)C)N(C)N1C3=CC=CC=C3
71	CGP 55845	no entry	O=P(CC1=CC=CC=C1)(C[C@@H](O)CN[C@H](C2=CC=C(Cl)C(Cl)=C2)C)O.Cl
72	Baicalin	no entry	O=C1C=C(C2=CC=CC=C2)OC3=C1C(O)=C(O)C(O[C@@H]4O[C@@H]([C@H]([C@@H]([C@H]4O)O)O)C(O)=O)=C3
73	Anisodamine	no entry	OCC(C1=CC=CC=C1)C(O[C@H]2C[C@@H]3C[C@@H]([C@@H](N3C)C2)O)=O
74	DU-14	no entry	O=S(OC1=CC=C(CCNC(CCCCCCCCCCCCC)=O)C=C1)(N)=O
75	Piperlonguminine	no entry	O=C(NCC(C)C)/C=C/C=C/C1=CC=C(OCO2)C2=C1
76	Roscovitine	no entry	CC(N1C=NC2=C(NCC3=CC=CC=C3)N=C(NC(CC)CO)N=C12)C
77	Palmatine	no entry	COC1=C(OC)C2=C[N+](CCC3=C4C=C(OC)C(OC)=C3)=C4C=C2C=C1.[Cl-]
78	Ipsapirone	no entry	O=C(C1=C2C=CC=C1)N(CCCCN3CCN(C4=NC=CC=N4)CC3)S2(=O)=O
79	Perospirone	no entry	O=C1N(CCCCN2CCN(C3=NSC4=C3C=CC=C4)CC2)C([C@]5([H])CCCC[C@]15[H])=O
80	Tandospirone	no entry	O=C1N(CCCCN2CCN(CC2)C3=NC=CC=N3)C([C@H]4[C@@H]1[C@H]5CC[C@@H]4C5)=O
81	RG-108	no entry	O=C(O)[C@@H](N1C(C(C=CC=C2)=C2C1=O)=O)CC3=CNC4=CC=CC=C43
82	Entinostat	no entry	NC1=CC=CC=C1NC(C2=CC=C(CNC(OCC3=CN=CC=C3)=O)C=C2)=O
83	Icariin	no entry	O=C1C(O[C@@H]2O[C@@H](C)[C@@H]([C@H]([C@H]2O)O)O)=C(C3=CC=C(OC)C=C3)OC4=C1C(O)=CC(O[C@@H]5O[C@@H]([C@H]([C@@H]([C@H]5O)O)O)CO)=C4CC=C(C)C
84	Yangonin	no entry	COC(C=C1)=CC=C1/C=C/C(O2)=CC(OC)=CC2=O
85	Epigallocatechin	no entry	O[C@@H]1CC2=C(C=C(C=C2O[C@H]1C3=CC(O)=C(C(O)=C3)O)O)O
86	Kavahin	no entry	COC1=CC(O[C@@H](/C=C/C2=CC3=C(OCO3)C=C2)C1)=O
87	Rotundine	no entry	COC1=C(C2=C(C[C@H]3C4=CC(OC)=C(OC)C=C4CCN3C2)C=C1)OC
88	Kaempferol	no entry	O=C(C1=C(O)C=C(O)C=C1O2)C(O)=C2C3=CC=C(O)C=C3
89	Pregnenolone	no entry	CC([C@H]1CC[C@H]2[C@@H]3CC=C4C[C@H](CC[C@@]4([C@H]3CC[C@]12C)C)O)=O
90	Resveratrol	no entry	OC1=CC=C(C=C1)/C=C/C2=CC(O)=CC(O)=C2
91	FG-4592	no entry	O=C(O)CNC(C1=C(O)C2=C(C(C)=N1)C=C(OC3=CC=CC=C3)C=C2)=O
92	PNU-120596	no entry	COC1=CC(OC)=C(C=C1NC(NC2=NOC(C)=C2)=O)Cl
93	TWS119	no entry	OC1=CC=CC(OC2=C3C(NC(C4=CC=CC(N)=C4)=C3)=NC=N2)=C1
94	Salidroside	no entry	OC[C@@H](O1)[C@@H](O)[C@H](O)[C@@H](O)[C@@H]1OCCC2=CC=C(O)C=C2
95	Cytidine	no entry	O=C1N=C(C=CN1[C@@H]2O[C@H](CO)[C@H]([C@H]2O)O)N

96	Piperine	no entry	O=C(/C=C/C=C/C1=CC2=C(OCO2)C=C1)N3CCCCC3
97	Pikamilone	no entry	O=C(NCCCC([O-])=O)C1=CC=CN=C1.[Na+]
98	EX-527	no entry	O=C([C@@H]1C(NC2=C3C=C(Cl)C=C2)=C3CCC1)N
99	Genistein	no entry	OC1=CC=C(C2=COC3=C(C2=O)C(O)=CC(O)=C3)C=C1
100	Daidzein	no entry	OC[C@H]1O[C@H]([C@@H]([C@H]([C@@H]1O)O)O)OC2=CC=C3C(C(C4=CC=C(C=C4)O)=COC3=C2)=O
101	UNC 0224	no entry	CN1CCC(NC2=C3C=C(OC)C(OCCCN(C)C)=CC3=NC(N4CCN(C)CCC4)=N2)CC1
102	IOX1	no entry	OC(C1=C2C=CC=NC2=C(C=C1)O)=O
103	Hydroxytacrine	no entry	NC1=C2C(O)CCCC2=NC3=CC=CC=C13.O=C(O)/C=C/C(O)=O
104	(R)-Baclofen	no entry	NC[C@@H](C1=CC=C(C=C1)Cl)CC(O)=O
105	Sulforaphane	no entry	CS(CCCCN=C=S)=O
106	Pikamilone	no entry	O=C(C1=CN=CC=C1)NCCCC(O)=O
107	Ampalex	no entry	O=C(N1CCCCC1)C2=CC=C3N=CC=NC3=C2
108	Sophoretin	no entry	OC1=CC2=C(C(C(O)=C(C3=CC=C(C(O)=C3)O)O2)=O)C(O)=C1
109	Sumanirole	no entry	CN[C@H]1CN2C3=C(NC2=O)C=CC=C3C1.OC(/C=C\C(O)=O)=O
110	UNC 0638	no entry	CC(N1CCC(NC2=C3C=C(OC)C(OCCCN4CCCC4)=CC3=NC(C5CCCCC5)=N2)CC1)C
111	UNC 0646	no entry	COC1=CC2=C(NC3CCN(C4CCCCC4)CC3)N=C(N5CCN(C(C)C)CCC5)N=C2C=C1OCCCN6CCCCC6
112	YC-5-169	no entry	O=C(CCCCCCC(CC1=C(N)C=CC=C1)=O)NC2=CC(C3=CN(C4=CC=CC=C4)N=N3)=CC=C2
113	Safinamide	no entry	O=C([C@H](C)NCC1=CC=C(OCC2=CC(F)=CC=C2)C=C1)N
114	CHIR-98014	no entry	NC1=NC(NCCNC2=NC=C(N3C=CN=C3)C(C4=CC=C(Cl)C=C4Cl)=N2)=CC=C1[N+]([O-])=O
115	IOX2	no entry	O=C(NCOOO)C1=C(O)C2=CC=CC=C2N(CC3=CC=CC=C3)C1=O
116	Blonanserin	no entry	FC1=CC=C(C2=CC(N3CCN(CC3)CC)=NC4=C2CCCCCC4)C=C1
117	Ganaxolone	no entry	C([C@H]1CC[C@@H]2[C@]1(C)CC[C@H]3[C@H]2CC[C@@H]4[C@]3(C)CC[C@@](C)(O)C4)=O
118	Harmane	no entry	CC1=NC=CC2=C1NC3=CC=CC=C32
119	Sazetidine A	no entry	OCCCCC#CC1=CC(OCC2CCN2)=CN=C1.OCCCCC#CC3=CC(OCC4CCN4)=CN=C3.Cl.Cl
120	AK-7	no entry	O=C(NC1=CC=CC(Br)=C1)C2=CC=CC(S(=O)(N3CCCCCC3)=O)=C2
121	PF 4778574	no entry	CC(S(=O)(N[C@@H]1[C@@H](C2=CC=C(C3=CC=C(C#N)S3)C=C2)COCC1)=O)C
