# Supplementary material for: High-throughput brain activity mapping and machine learning as a foundation for systems neuropharmacology
Source: Nat Commun. 2018 Dec 3;9:5142. doi: 10.1038/s41467-018-07289-5 (PMC6277389; doi:10.1038/s41467-018-07289-5)
Supplement: Supplementary file 7 — Reporting Summary [file 41467_2018_7289_MOESM7_ESM.pdf]

nature research

Corresponding author(s): Xin Wang, Stephen J. Haggarty, Peng Shi

Reporting Summary

Nature Research wishes to improve the reproducibility of the work that we publish. This form provides structure for consistency and transparency in reporting. For further information on Nature Research policies, see Authors & Referees and the Editorial Policy Checklist.

Statistical parameters

When statistical analyses are reported, confirm that the following items are present in the relevant location (e.g. figure legend, table legend, main text, or Methods section).

n/a	Confirmed	
☐	☒	The exact sample size (n) for each experimental group/condition, given as a discrete number and unit of measurement
☐	☒	An indication of whether measurements were taken from distinct samples or whether the same sample was measured repeatedly
☐	☒	The statistical test(s) used AND whether they are one- or two-sided<br>*Only common tests should be described solely by name; describe more complex techniques in the Methods section.*
☐	☒	A description of all covariates tested
☐	☒	A description of any assumptions or corrections, such as tests of normality and adjustment for multiple comparisons
☐	☒	A full description of the statistics including central tendency (e.g. means) or other basic estimates (e.g. regression coefficient) AND variation (e.g. standard deviation) or associated estimates of uncertainty (e.g. confidence intervals)
☐	☒	For null hypothesis testing, the test statistic (e.g. F, t, r) with confidence intervals, effect sizes, degrees of freedom and P value noted<br>*Give P values as exact values whenever suitable.*
☒	☐	For Bayesian analysis, information on the choice of priors and Markov chain Monte Carlo settings
☒	☐	For hierarchical and complex designs, identification of the appropriate level for tests and full reporting of outcomes
☒	☐	Estimates of effect sizes (e.g. Cohen's d, Pearson's r), indicating how they were calculated
☐	☒	Clearly defined error bars<br>*State explicitly what error bars represent (e.g. SD, SE, CI)*

Our web collection on statistics for biologists may be useful.

Software and code

Policy information about availability of computer code

Data collection	Micro-manager 1.4.22; EthoVision Xt.
Data analysis	Matlab (9.2); ImageJ (1.51u); R (3.4.3).

For manuscripts utilizing custom algorithms or software that are central to the research but not yet described in published literature, software must be made available to editors/reviewers upon request. We strongly encourage code deposition in a community repository (e.g. GitHub). See the Nature Research guidelines for submitting code & software for further information.

Data

Policy information about availability of data

All manuscripts must include a data availability statement. This statement should provide the following information, where applicable:

- Accession codes, unique identifiers, or web links for publicly available datasets
- A list of figures that have associated raw data
- A description of any restrictions on data availability

The data that support the finding of this study is available from the corresponding author upon request.

Field-specific reporting

Please select the best fit for your research. If you are not sure, read the appropriate sections before making your selection.

☒ Life sciences ☐ Behavioural & social sciences ☐ Ecological, evolutionary & environmental sciences

For a reference copy of the document with all sections, see nature.com/authors/policies/ReportingSummary-flat.pdf

Life sciences study design

All studies must disclose on these points even when the disclosure is negative.

Sample size	5 larvae were examined in the screening based on brain activity mapping, 12 larvae were used in the behavioral tests. For the screening part, 179 clinical drugs and 121 non-clinical compounds were included in the training and testing set. These information has been reported in the manuscript.
Data exclusions	No sample were excluded from analysis.
Replication	Several replications were performed to confirm our findings. First, to confirm background level brain activity maps, we acquired the BAMs in 50 zefrafish lavae, randomly select 5 (out of 50) to calculate the T-score BAM, and repeat this process for 1000 times to get the distribution of the background level of T-score BAMs (Supplementary Fig. 3). Second, we randomly selected subgroup of 20 compounds from the test set, and repeated the BAM experiments (5 larvae for each compound) to test the reliability of our strategy (Supplementary Fig. 14). Third, we tested 7 extra clinical anti-epileptic drugs that were not in the training or test set to use as a positive control experiment (Supplementary Fig. 15 and Supplementary Table 2).
Randomization	No randomization is needed. We used only one type of animal, and the screening were conducted blindly.
Blinding	For the initial screening in this study, all details about the compounds' structure, therapeutic functions, mechanisms of actions and names were blind to the operator and analyzer.

Reporting for specific materials, systems and methods

Materials & experimental systems

n/a	Involved in the study
☒	☐ Unique biological materials
☒	☐ Antibodies
☒	☐ Eukaryotic cell lines
☒	☐ Palaeontology
☐	☒ Animals and other organisms
☒	☐ Human research participants

Methods

n/a	Involved in the study
☒	☐ ChIP-seq
☒	☐ Flow cytometry
☒	☐ MRI-based neuroimaging

Animals and other organisms

Policy information about studies involving animals; ARRIVE guidelines recommended for reporting animal research

Laboratory animals	Transgenic zebrafish line elavl3:GCaMP5G, Larvae of 6-8 dpf were used.
Wild animals	n.a.
Field-collected samples	The zebrafish line was maintained in aquaria under standard laboratory conditions (at 28 ˚C under a cycle of 14 h light, 10 h dark). After experiments, specimens were kept in a Petri dish for sevral days for normal development.
